# Transcriptome and metabolome comprehensive analysis reveal the molecular basis of slow-action and non-repellency of cycloxaprid against an eusocial pest, *Solenopsis invicta*

**DOI:** 10.3389/fphys.2023.1274416

**Published:** 2023-11-27

**Authors:** Chengju Du, Kaibin Jiang, Zhiping Xu, Lei Wang, Jie Chen, Cai Wang

**Affiliations:** ^1^ College of Forestry and Landscape Architecture, South China Agricultural University, Guangzhou, China; ^2^ Guangdong Provincial Key Laboratory of Silviculture Protection and Utilization, Guangdong Academy of Forestry, Guangzhou, China; ^3^ Shanghai Key Laboratory of Chemical Biology, School of Pharmacy, East China University of Science and Technology, Shanghai, China; ^4^ College of Plant Protection, South China Agricultural University, Guangzhou, China; ^5^ Guangdong Provincial Key Laboratory of High Technology for Plant Protection, Institute of Plant Protection, Guangdong Academy of Agricultural Sciences, Guangzhou, China

**Keywords:** eusocial insect, formicidae, red imported fire ant, neonicotinoid insecticide, cycloxaprid

## Abstract

The eusocial pest, red imported fire ant (*Solenopsis invicta*), is a highly invasive species that poses significant threats to public safety, agriculture, and the ecological environment. Cycloxaprid, a newly identified effective, slow-acting, and non-repellent insecticide against *S. invicta*, allows contaminated individuals to transfer the insecticide among nestmates through body contact. However, the molecular-level changes occurring in *S. invicta* post cycloxaprid exposure and any molecular alterations contributing to the slow demise or decreased sensitivity remain unclear. In this study, transcriptomic and metabolomic techniques were used to investigate the molecular mechanisms of *S. invicta* exposed to cycloxaprid. Differential analysis results revealed 275, 323, and 536 differentially expressed genes at 12, 24, and 48 h, respectively. Genes involved in lipid and energy metabolism, DNA integration, and hormone synthesis were largely upregulated at 12 h, suggesting *S. invicta* might actively resist cycloxaprid impacts, and predominantly downregulated at 48 h, indicating further functional impairment and impending death. Also, we observed an imbalance in olfactory perception pathways at 12 h, which may indicate a disruption in the olfactory system of *S. invicta*. Metabolomic results showed that the regulation of most differential metabolites (DMs) was consistent with the expression changes of their related DEGs at different time points. Our study provides insights into the mechanism underlying slow-acting and non-repellent properties of cycloxaprid against *S. invicta*.

## 1 Introduction

The red imported fire ant, *Solenopsis invicta*, is a highly destructive eusocial pest that has invaded more than 20 countries and regions worldwide, including the United States, Australia, and China (Ascunce et al., 2011; Wetterer, 2013). This pest poses significant threats to public safety, agriculture, and the ecological environment (Gutrich et al., 2007; Vinson, 2013; Wang et al., 2020). Medically, *S*. *invicta* stings can cause intense pain, blistering, pustules, allergic reactions, and, in extreme cases, anaphylactic shock or fatal outcomes (Haddad Junior and Larsson, 2015; Bernaba et al., 2019). Also, *S*. *invicta* can cause damage to the roots and seeds of various crops, leading to reduced yields and economic losses in agricultural systems (Chan and Guénard, 2020). Additionally, the invasion of *S. invicta* significantly deceased the abundance and diversity of native ants and other arthropods (Morrison, 2002). Given these issues, *S. invicta* resulted in massive global economic costs for damage and management (Angulo et al., 2022). For example, *S. invicta* was estimated to lead to annual costs of USD 6 billion in the United States and AUD 1.65 billion in Australia (Pereira et al., 2002; Wylie and Janssen-May, 2017).

Eusocial insects exhibit complex behaviors and biological features (e.g., highly organized social structures, hierarchical communication systems, task specialization, and unique food-sharing behaviors) that differentiate them from solitary insects (Robinson, 1992; Gordon, 2019). These characteristics not only ensure the high adaptability and flexibility of eusocial pests to invade diverse habitats but also pose challenges to their control. Traditional insecticides can rapidly eliminate individuals of eusocial pests outside the nest, but they cannot easily reach the reproducers and larvae concealed deep within the nest, resulting in inadequate control at a colony level (Drees and Gold, 2003; Silverman and Brightwell, 2008; Rust and Su, 2012). Therefore, insecticides to control eusocial pests such as *S. invicta* should be slow-acting and non-repellent to ensure the horizontal transfer of insecticides among nestmates through physical contact and eventually eradicate the whole colony. Currently, insecticides such as imidacloprid, fipronil, rotenone, and spinosad have been widely applied for *S. invicta* management (Chen and Oi, 2020).

Cycloxaprid is a novel oxabridged *cis*-nitromethylene neonicotinoid insecticide characterized by a nitro (-NO_2_) group in a *cis*-configuration (Shao et al., 2013a). This insecticide appears to activate a different site compared to traditional neonicotinoid insecticide on the nicotinic acetylcholine receptors (nAChRs) and exhibits high insecticidal activity (Shao et al., 2013b; Zhang et al., 2019). For now, it has been applied to control various pests, including *A. gossypii*, *Bemisia tabaci*, and *Sitobion avenae* (Cui et al., 2016; Wang et al., 2018a; Cui et al., 2020). For example, Cui et al. (2012) reported that cycloxaprid exhibited intense contact and root-systemic activity against *S. avenae*, negatively affecting its feeding behavior and growth rate. Also, exposure to sublethal levels of cycloxaprid significantly shortened the pre-oviposition period and life expectancy of *Aphis gossypii* and inhibited the activity of various detoxification enzymes (Cui et al., 2018).

Our previous studies showed that cycloxaprid is a slow-acting and non-repellent insecticide against *S. invicta* workers at certain concentrations (Zhang et al., 2022). For example, surfaces treated with cycloxaprid (0.3 μg/cm^2^) led to high mortality in *S. invicta* workers. However, cycloxaprid caused a much slower speed of kill than other fire ant control agents such as bifenthrin. In addition, cycloxaprid did not significantly influence the foraging, digging, and nesting behaviors of *S. invicta* workers. Interestingly, both corpses and live ants previously exposed to cycloxaprid caused horizontal toxicity against untreated ants. However, the molecular-level changes within *S. invicta* post cycloxaprid exposure remain unclear. In this study, we analyzed changes in gene expression and metabolites in *S. invicta* workers after exposure to cycloxaprid for 12, 24, and 48 h. By examining the differences in genes and metabolites, we aimed to reveal the mechanism of action of cycloxaprid on *S. invicta* and discuss the potential reasons underlying its slow-acting and non-repellent properties.

## 2 Materials and methods

### 2.1 Ant collection and maintenance

Six *S. invicta* colonies were collected from Tianlu Lake Forest Park (23°12′ N, 113°8′ E), Guangzhou, China, on 3 November 2021. Mounds were more than 5 m apart at the collection site. Nest materials with immatures (eggs, larvae, and pupae) and adults (workers and queens) of *S. invicta* were rapidly shoveled into a plastic container (length: 50 cm, width: 37 cm, height: 26 cm; Kangyi Co., Ltd., Hubei, China). The walls of the containers were previously smeared with talcum powder, and therefore *S. invicta* cannot climb and escape (Ning et al., 2019). The collected *S. invicta* colonies were brought to the laboratory and reared under relatively constant conditions (a photoperiod of 14 h light: 10 h dark, and room temperature of 21°C–23°C). Ten days before the experiment, *S. invicta* colonies were extracted from the soil using the water-dropping method provided by Chen (2007). The extracted ants were transferred to another plastic container (the inner walls were smeared with the talcum powder as mentioned above). Frozen crickets, water, and 20% honey water were supplied.

### 2.2 Cycloxaprid treatment

Cycloxaprid was obtained from the School of Pharmacy, East China University of Science and Technology, Shanghai, China. Plastic boxes (length: 44 cm, width: 29 cm, height: 19 cm; Kangyi Co., Ltd., Hubei, China) with talcum powder-coated inner walls were used as experimental arenas. Cycloxaprid was dissolved in acetone (analytical reagent, Fuyu Chemical Co., Ltd., Tianjin, China) to prepare for the aimed concentrations. The required amount of solution was then evenly smeared onto the bottom of the boxes to reach the cycloxaprid concentration of 0.3 μg/cm^2^ after complete evaporation of the acetone. The same amount of acetone (without cycloxaprid) was added to the control boxes. For each colony, 10 g of *S. invicta* workers were weighed and transferred to a cycloxaprid-treated or untreated box, and 10-mL tubes containing water or 20% honey water were supplied. Here, we only tested worker ants because the immatures and reproductive castes (i.e., queens) may have different molecular responses that may interfere with the results of workers (Kohlmeier et al., 2017; Negroni et al., 2021). Each box was placed in a larger plastic container (length: 50 cm, width: 37 cm, height: 26 cm), where moistened paper towels were placed on the bottom to maintain humidity (Wen et al., 2022). The bioassays were maintained at room temperature (21°C–23°C). At 12, 24, and 48 h, 500 mg of live *S. invicta* workers were randomly collected from each experimental box and immediately transferred to liquid nitrogen, then stored in a −80°C freezer.

### 2.3 Transcriptomic analysis

The transcriptome of *S. invicta* workers collected from cycloxaprid-treated or untreated boxes was analyzed using RNA-seq. *Solenopsis invicta* colonies 1-3 were used in this test, with each colony as an independent biological replicate. In the sample preparation process, 20 mg *S. invicta* workers were pulverized into a fine powder under liquid nitrogen. Subsequently, the total RNA was isolated using TRIzol reagent (Invitrogen, USA), adhering strictly to the manufacturer’s guidelines. The RNA purification, library construction, and sequencing were carried out utilizing the Illumina sequencing platform, with all services provided by Metware Biotechnology Co., Ltd. (Wuhan, China). We employed Fastp (v0.19.3) to filter out low-quality reads and adapter sequences (Chen et al., 2018). We also utilized HISAT (v2.1.0) to construct the index and align clean reads with the reference genome (https://www.ncbi.nlm.nih.gov/assembly/GCF_016802725.1/#/st) (Kim et al., 2015). StringTie (v1.3.4d) was adopted to predict new genes (Pertea et al., 2016), and the transcripts were annotated using Diamond (v2.1.8) (Buchfink et al., 2015). The task of counting aligned reads and calculating the Fragments Per Kilobase of transcript per Million (FPKM) of each gene according to the gene length was performed using FeatureCounts (v1.6.2) (Liao et al., 2014). The differential expression between cycloxaprid-treated and untreated ants was analyzed by utilizing DESeq2 (v1.22.1) (Love et al., 2014) and EdgeR (Robinson et al., 2010), with *p* values corrected through the Benjamini–Hochberg method (Haynes, 2013). To identify differentially expressed genes (DEGs), a threshold of |log2foldchange| (log2FC) > 1 and a *p*-value < 0.05 were applied as the markers of significant differential expression.

The functional analyses with gene annotation and enrichment analyses were conducted to investigate the functions of DEGs at different time points. DEGs were annotated using the Gene Ontology (GO), Kyoto Encyclopedia of Genes and Genomes (KEGG), Non-Redundant Protein Sequence Database (NR), Kentucky Online Gateway (KOG), Swiss-Prot, and Pfam databases. We executed enrichment analysis using the hypergeometric test. In the case of KEGG, the test was carried out at the pathway level, whereas for GO, the testing was based on the specific GO term.

### 2.4 Quantitative real-time PCR

To ascertain the precision and dependability of the transcriptome, we conducted an analysis using qRT-PCR on the total RNA of samples previously treated or untreated with cycloxaprid. *Solenopsis invicta* colonies 1-3 were tested, with each colony as an independent biological replicate. Eight selected genes (*AASS*, *ACO*, *ORAI*, *SCD*, *CYP450*-1, *CYP450*-2, *CYP450*-3, *GST*) were chosen based on their significant differential expression (see results) as well as their important roles in key biological processes (i.e., fatty acid synthesis and metabolism [*AASS*, *ACO*, *SCD*], energy conversion [*ORAI*], and detoxification [*CYP450*, *GST*]). We employed Ribosomal protein S3 (*RPS3*; XM_011172385) as the internal reference gene for normalization (Chen et al., 2022). Specific gene primer pairs were crafted utilizing the NCBI Primer-BLAST tool (http://www.ncbi.nlm.nih.gov/tools/primer-blast/). The primers used in this study are detailed in Supplementary Table S1. Each sample’s total RNA (2 µg) was transcribed in reverse to cDNA utilizing HiScript III RT SuperMix for qPCR (+gDNA wiper) (Vazyme, Nanjing, China), adhering strictly to the manufacturer’s guidelines. Subsequently, the qRT-PCR process was executed employing the Option Real-Time PCR System CFX96 (Bio-Rad, CA). The procedure conditions comprised an initial pre-denaturation phase at 95°C lasting for 2 min, followed by 39 cycles of denaturation at 95°C for 5 s and annealing at 60°C for 30 s. Following this, a melting curve was created by gradually escalating the temperature from 65°C to 95°C. Each qPCR operation was carried out in triplicate as three technical replicates to assess the variability introduced by the experimental procedure. The 2^−ΔΔCT^ method (Livak and Schmittgen, 2001) was employed to determine the relative expression levels of the target genes. Statistical scrutiny was undertaken using a paired *t*-test, with a *p*-value < 0.05 indicating the statistical significance.

### 2.5 Metabolomic analysis

The metabolome of *S. invicta* workers collected from cycloxaprid-treated or untreated boxes was analyzed using Widely-targeted metabolomic analysis (Chen et al., 2013). Six *S. invicta* colonies were tested, each as an independent biological replicate. Samples kept at −80°C were defrosted on ice and then pulverized using liquid nitrogen. A solution of 400 μL, comprising methanol and water in a ratio of 7:3, was blended with an internal standard and added to the 20 mg of the crushed sample. This composite was then agitated at 1,500 rpm for 5 min. Following a 15-min icing period, the samples were centrifuged at 12,000 rpm for 10 min at 4°C. A portion of the supernatant (300 μL) was set aside and cooled at −20°C for 30 min, then centrifuged at 12,000 rpm for 3 min at 4°C. For Liquid chromatography-mass spectrometry (LC-MS) analysis, a 200 μL aliquot of the supernatant was processed using the Ultra-Performance Liquid Chromatography and Tandem Mass Spectrometry (UPLC-MS/MS) systems provided by Wuhan Metware Biotechnology Co., Ltd. (China).

To identify Differential metabolites (DMs), a threshold of |log2foldchange| (log2FC) > 1 and a VIP >1 were applied as the markers of significant difference. VIP values were derived from Partial least squares discriminant analysis (PLS-DA) outcomes using the MetaboAnalystR package in R. Before PLS-DA, the data underwent log-transformation and mean-centering. A permutation test involving 200 permutations was implemented to prevent overfitting. The R function ‘plsr’ was employed to perform unsupervised PLS-DA, following a unit variance-scaling of the data. Annotation of DMs was accomplished through the KEGG Compound database, with mapping to the KEGG Pathway database. Statistically significant enrichment pathways were identified using the *p*-value from a hypergeometric test for a specified list of metabolites.

### 2.6 Correlation analysis of transcriptomic and metabolomic data

A correlation analysis was carried out between transcriptome and metabolome to gain deeper insight into the regulatory network of *S. invicta* workers responding to cycloxaprid. The transcriptomic and metabolomic analysis data from samples of colonies 1-3 were used for correlation analysis. The Pearson Correlation Coefficient (PCC) for differentially expressed genes and metabolites was computed employing the ‘cor’ function within the R package. To showcase the fold changes in genes and metabolites across different groups, a nine-quadrant plot was generated with a PCC >0.6 and *p*-value <0.05, using the ggplot2 and getopt packages in R. For generating a correlation network diagram, genes and metabolites with a PCC >0.85 and a *p*-value <0.05 were chosen, using Cytoscape (v3.9.1) as per Shannon et al. (2003).

## 3 Result

### 3.1 Transcriptomic analysis

**FIGURE 1:**
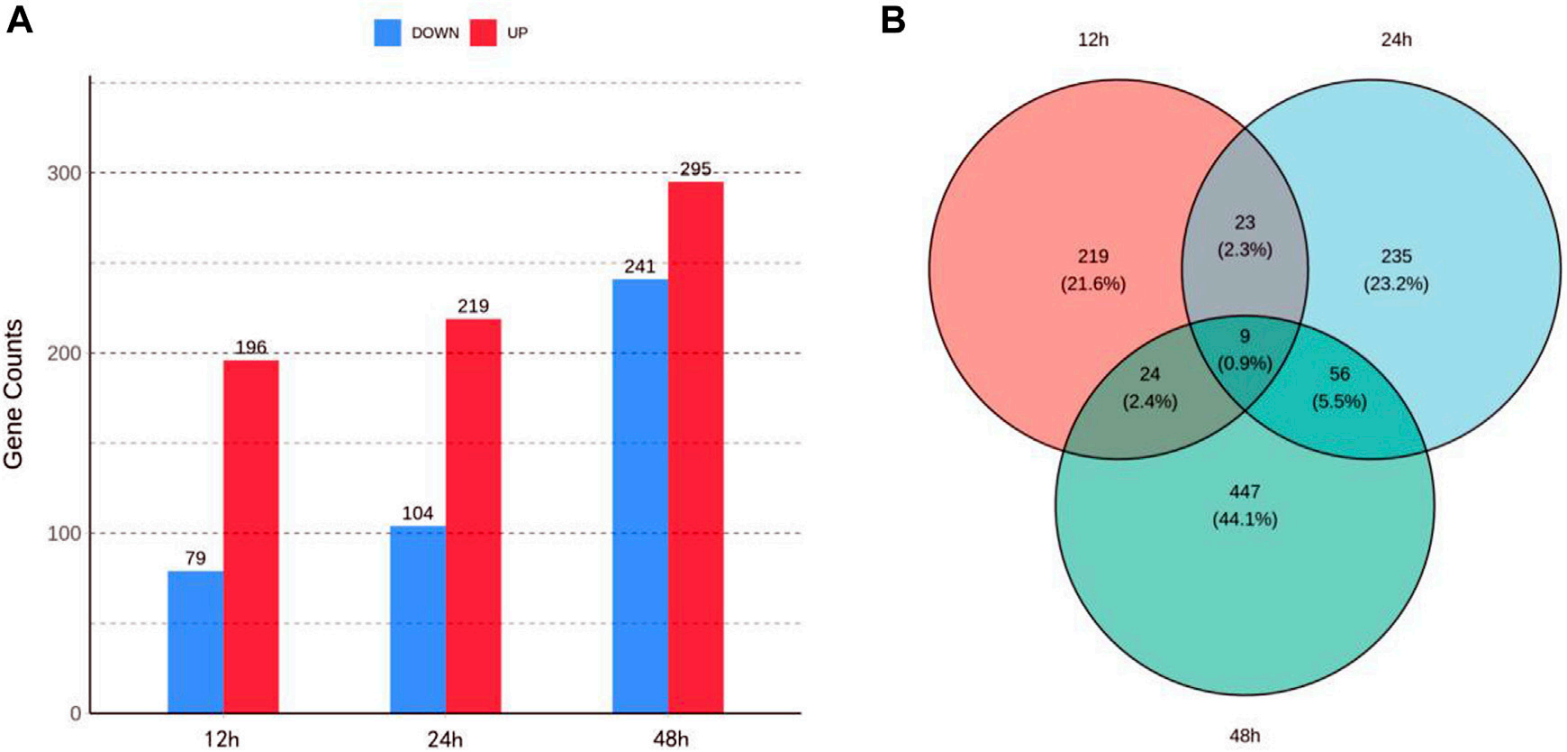
Transcriptomic analysis of *Solenopsis invicta* at three time points post cycloxaprid exposure. **(A)** The number of upregulated and downregulated differentially expressed genes (DEGs) at three time points; **(B)** Venn diagram illustrating the overlap of DEGs at the three time points, highlighting the shared and unique responses to cycloxaprid exposure.

Each sample yielded 42,596,782 to 56,609,672 clean reads, with Q30 percentages ranging from 90.38% to 92.81% and GC content ranging from 44.29% to 48.15% (Supplementary Table S2), reflecting the high quality of the transcriptome sequencing data. Following the alignment of clean reads to the reference genome of *S. invicta*, we identified 15,673 to 15,967 expressed genes for each sample. A total of 275, 323, and 536 DEGs at 12, 24, and 48 h were identified, respectively, using a threshold of |log2FC| > 1 and *p*-value < 0.05 (Figure 1A). Venn diagram (Figure 1B) revealed 23 shared DEGs between 12 and 24 h, 24 shared DEGs between 12 and 48 h, 56 shared DEGs between 24 and 48 h, and 9 shared DEGs across all three time points.

**FIGURE 2:**
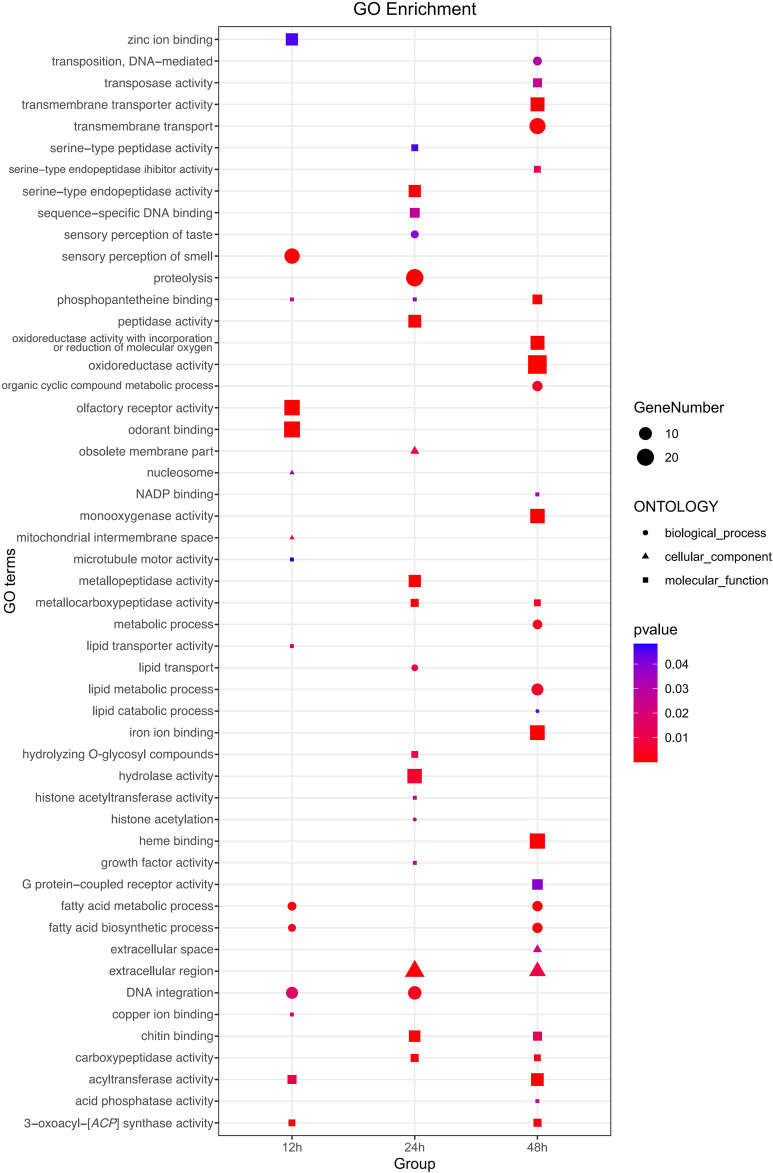
Comprehensive Gene Ontology (GO) enrichment analysis of DEGs at three distinct time points post cycloxaprid exposure. The size of each dot represents the number of genes involved in the corresponding GO term, while the shape symbolizes the specific functional classification of the respective GO term. *p*-values, calculated using hypergeometric tests, demonstrate the statistical significance of the enrichment results.

**FIGURE 3:**
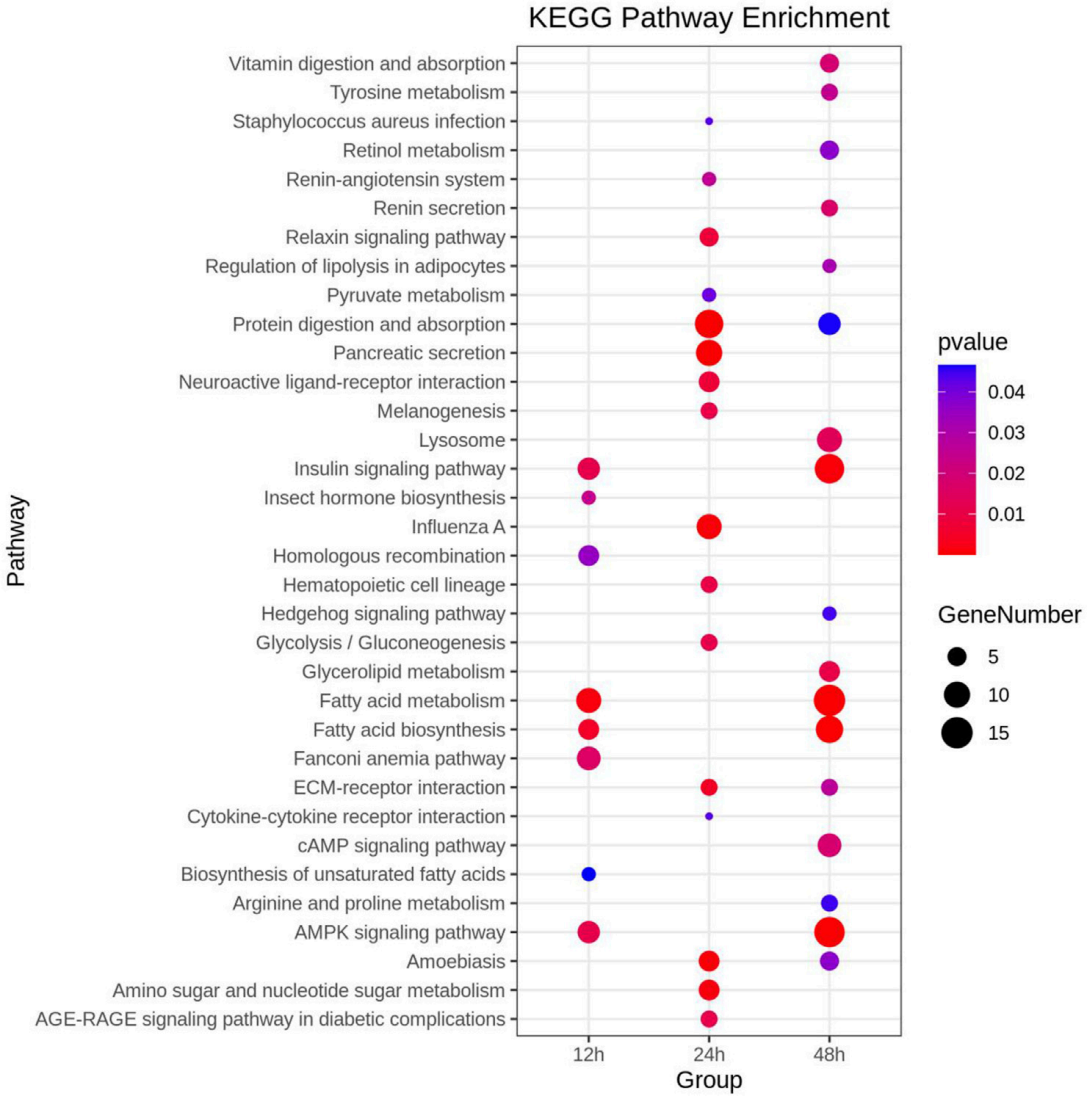
Comprehensive Kyoto Encyclopedia of Genes and Genomes (KEGG) enrichment analysis of DEGs at three distinct time points post cycloxaprid exposure. The size of each dot represents the number of genes involved in the corresponding KEGG pathways. *p*-values, calculated using hypergeometric tests, demonstrate the statistical significance of the enrichment results.

Functional analyses provided insights into significantly enriched pathways (Figure 2; Figure 3) and the involvement of upregulated and downregulated DEGs in these pathways (Supplementary Figure S1, S2). At 12 h, the significantly enriched pathways primarily consisted of upregulated DEGs associated with fatty acid metabolism, fatty acid biosynthesis, AMPK signaling, insulin signaling, acyltransferase activity, DNA integration, and insect hormone biosynthesis. At 24 h, the significantly enriched pathways also primarily comprised upregulated DEGs, which were involved in protein digestion and absorption, pancreatic secretion, proteolysis, extracellular region, and amino sugar and nucleotide sugar metabolism. At 48 h, the significantly enriched pathways included both upregulated and downregulated DEGs. Specifically, DEGs involved in fatty acid metabolism, fatty acid biosynthesis, AMPK signaling pathway, insulin signaling pathway, iron ion binding, and heme binding were downregulated. In contrast, some DEGs involved with transmembrane transport, extracellular region, and cAMP signaling were upregulated. In addition, 22 DEGs involved in sensory perception of smell, odorant binding, and olfactory receptor activity displayed an inconsistent distribution at 12 h, with 13 upregulated and 9 downregulated.

### 3.2 Quantitative real-time PCR

**FIGURE 4:**
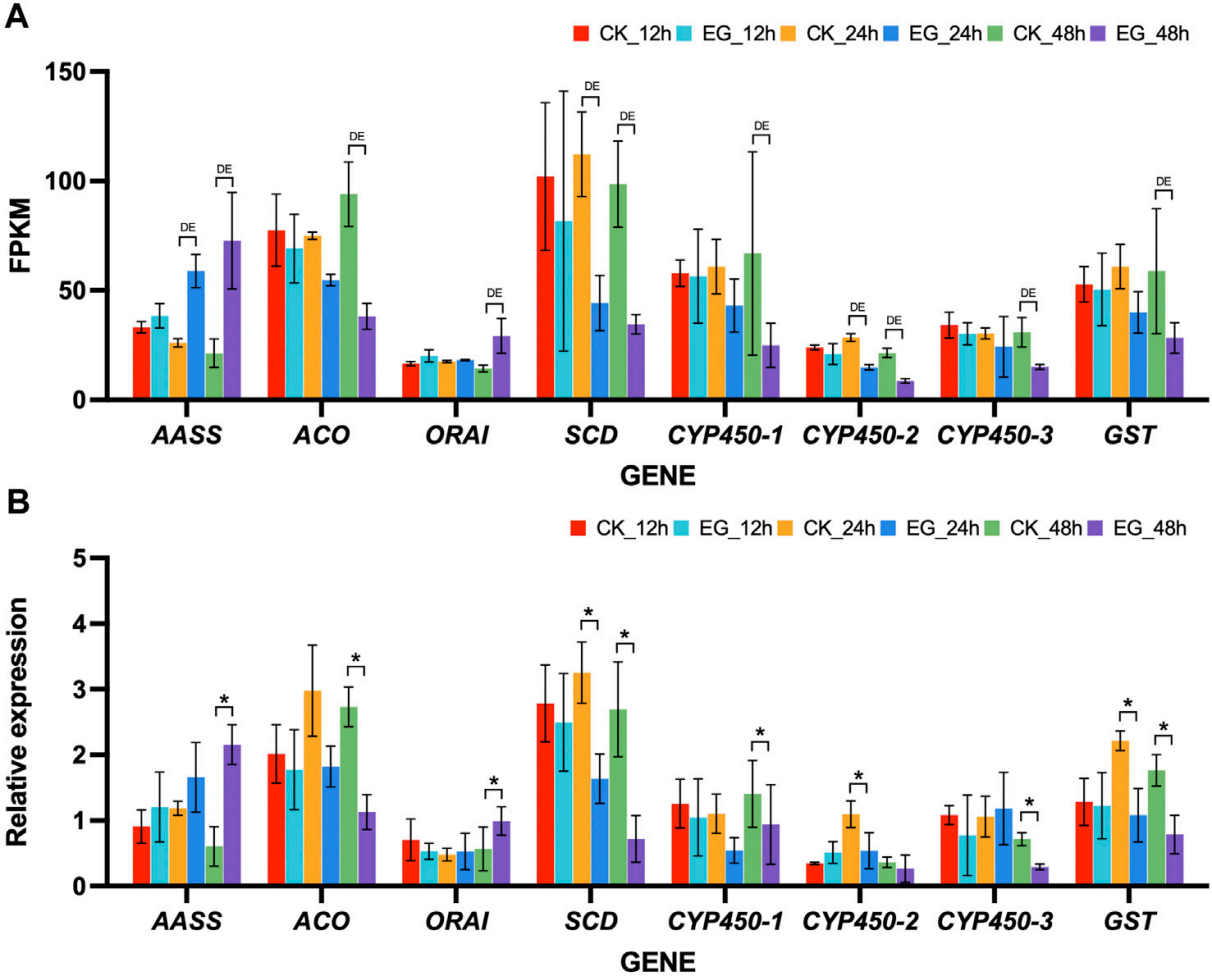
Expression patterns of 8 selected DEGs in *Solenopsis invicta* across three time points post cycloxaprid exposure. **(A)** Expression levels of the selected genes based on Fragments Per Kilobase of transcript per Million (FPKM) values obtained from RNA-seq data. “DE” denotes differential expression, which has been validated through the use of “DESeq2” or “EdgeR” tests, indicating a statistically significant difference in gene expression between the control and experimental groups at the corresponding time point. **(B)** Relative expression levels of the selected genes were obtained via quantitative real-time PCR (qRT-PCR) analysis. Error bars represent the standard error of the mean (SEM) from three replicates. “*” marks instances where the paired *t*-test yielded a *p*-value <0.05, suggesting a significant difference in gene expression between the control and experimental groups at the corresponding time point. CK and, EG indicate the control group and the experimental group (fire ants treated with cycloxaprid), respectively, followed by the time point of sampling. The consistency between the RNA-seq and qRT-PCR data highlights the reliability of our transcriptomic analysis.

Accordance was observed between the qRT-PCR expression patterns and the FPKM values valued from RNA-seq data (Figure 4). At specific time points, statistical analysis revealed significant differences (*p*-value < 0.05) in the expression levels of selected genes between cycloxaprid-treated and untreated ants. Specifically, *AASS* and *ORAI* were significantly upregulated at 48 h; *CYP450*-2 was significantly downregulated at 24 h; *ACO*, *CYP450*-1, and *CYP450*-3 were significantly downregulated at 48 h; *SCD* and *GST* were significantly downregulated at 24 h and 48 h. The consistency of the RNA-seq and qRT-PCR data underscored the precision and dependability of our transcriptomic analysis.

### 3.3 Metabolomic analysis

**FIGURE 5:**
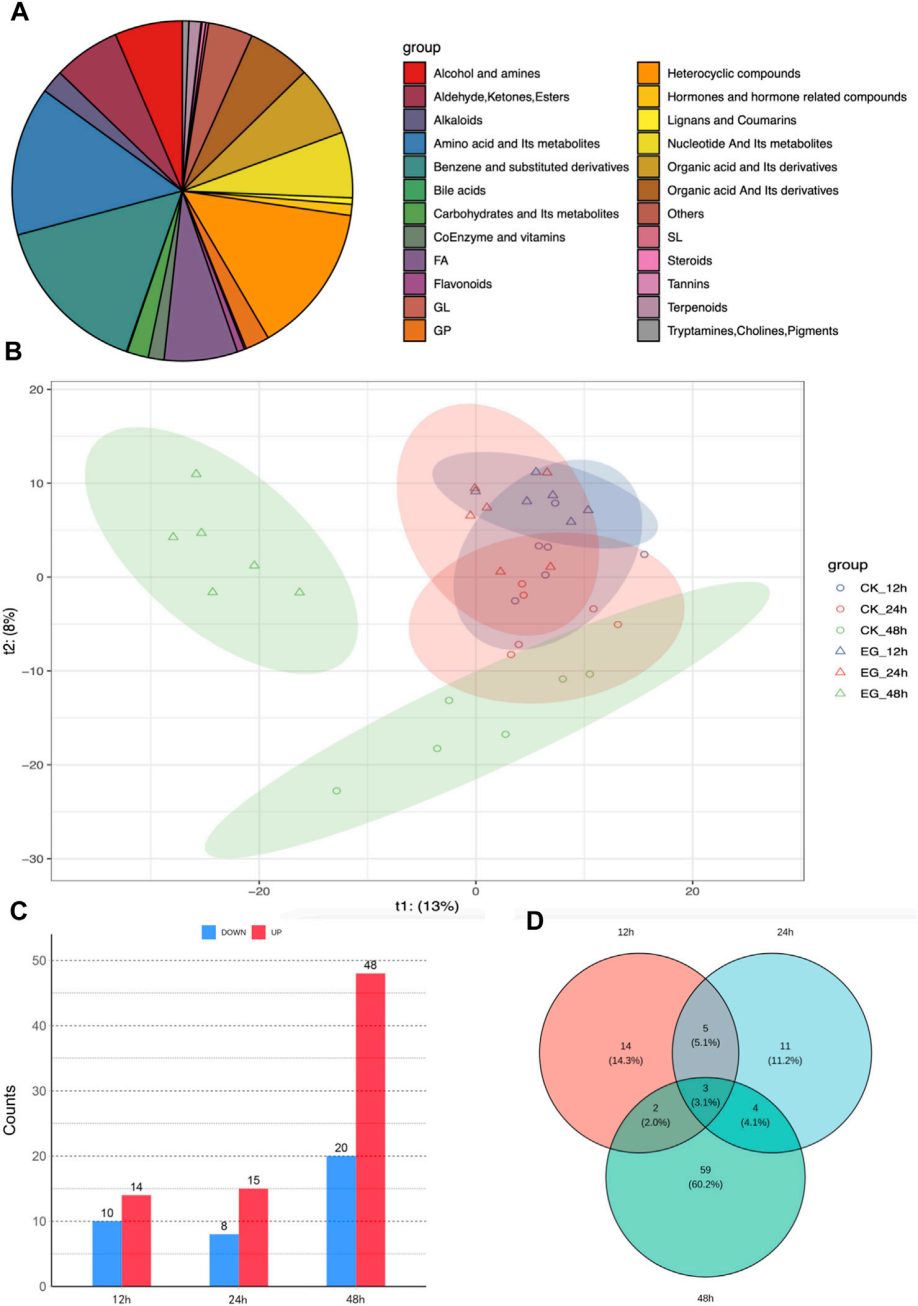
Metabolomic analysis of *Solenopsis invicta* at three time points post cycloxaprid exposure. **(A)** Classification of detected metabolites; **(B)** Partial Least Squares Discriminant Analysis (PLS-DA) results for six sample groups, illustrating the progressive separation between the experimental and control groups across the three time points; **(C)** The number of upregulated and downregulated differentially expressed metabolites (DMs) at three time points; **(D)** Venn diagram illustrating the overlap of DMs at the three time points, highlighting the shared and unique responses to cycloxaprid exposure.

UPLC-MS/MS detected 24 classes and 1,135 metabolites (Figure 5A). The PLS-DA analysis results demonstrated a progressive separation between the experimental and control groups across the three time points (12, 24, and 48 h). The separation gradually became more evident as the exposure time increased, and by 48 h, the clustering was distinctly clear (Figure 5B). A total of 24, 23, and 68 DMs at 12, 24, and 48 h were identified, respectively, using a threshold of |log2FC| > 2 and VIP >0.05 (Figure 5C). Venn diagram (Figure 5D) revealed 5 shared DEGs between 12 and 24 h, 2 shared DEGs between 12 and 48 h, 4 shared DEGs between 24 and 48 h, and 3 shared DMs across all three time points.

**FIGURE 6:**
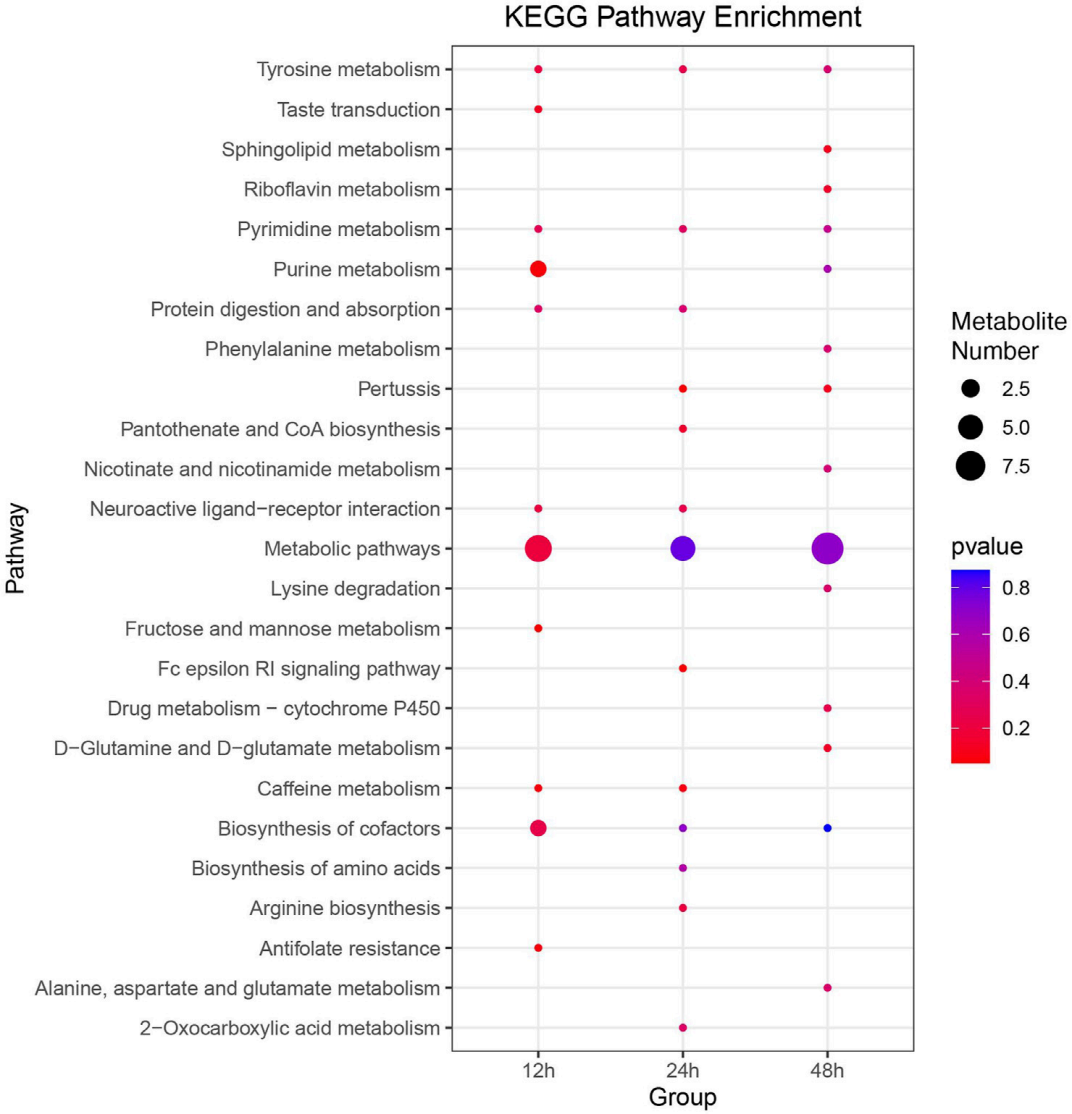
Comprehensive KEGG enrichment analysis of DMs at three distinct time points post cycloxaprid exposure. The size of each dot represents the number of genes involved in the corresponding KEGG pathways. *p*-values, calculated using hypergeometric tests, demonstrate the statistical significance of the enrichment results.

Utilizing the KEGG database, 346 out of 1,135 metabolites were annotated. Additionally, KEGG enrichment analysis revealed that DMs were enriched in 11, 13, and 15 pathways at 12, 24, and 48 h, respectively (Figure 6). Pathways, including metabolic pathways, neuroactive ligand-receptor interaction, biosynthesis of cofactors, and tyrosine metabolism, were significantly enriched at all three time points. Differential abundance score plots illustrated the changing trends of enriched pathways (Supplementary Figure S3–S5). At 12 h, DMs in the fructose and mannose metabolism and antifolate resistance were upregulated, whereas DMs in the caffeine metabolism were downregulated. At 24 h, DMs in the pertussis and Fc epsilon RI signaling pathway were upregulated, whereas DMs in the caffeine metabolism were downregulated. At 48 h, DMs in the pertussis and riboflavin metabolism were upregulated, whereas DMs in the sphingolipid metabolism and D-Glutamine and D-glutamate metabolism were downregulated.

### 3.4 Correlation analysis of the transcriptomic and metabolomic data

**FIGURE 7:**
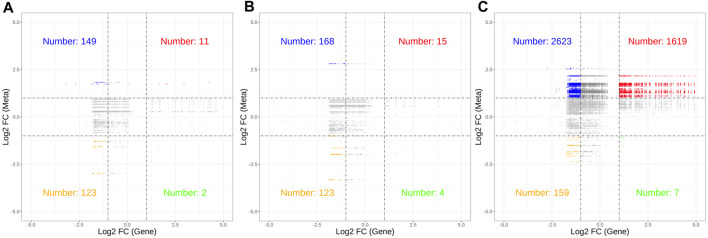
Nine-quadrant diagrams showed correlations between genes and metabolites in *Solenopsis invicta* at 12 **(A)**, 24 **(B)**, and 48 h **(C)** post cycloxaprid exposure. These diagrams display dynamic relationships between gene expression and metabolic changes in response to cycloxaprid exposure over time.

**FIGURE 8:**
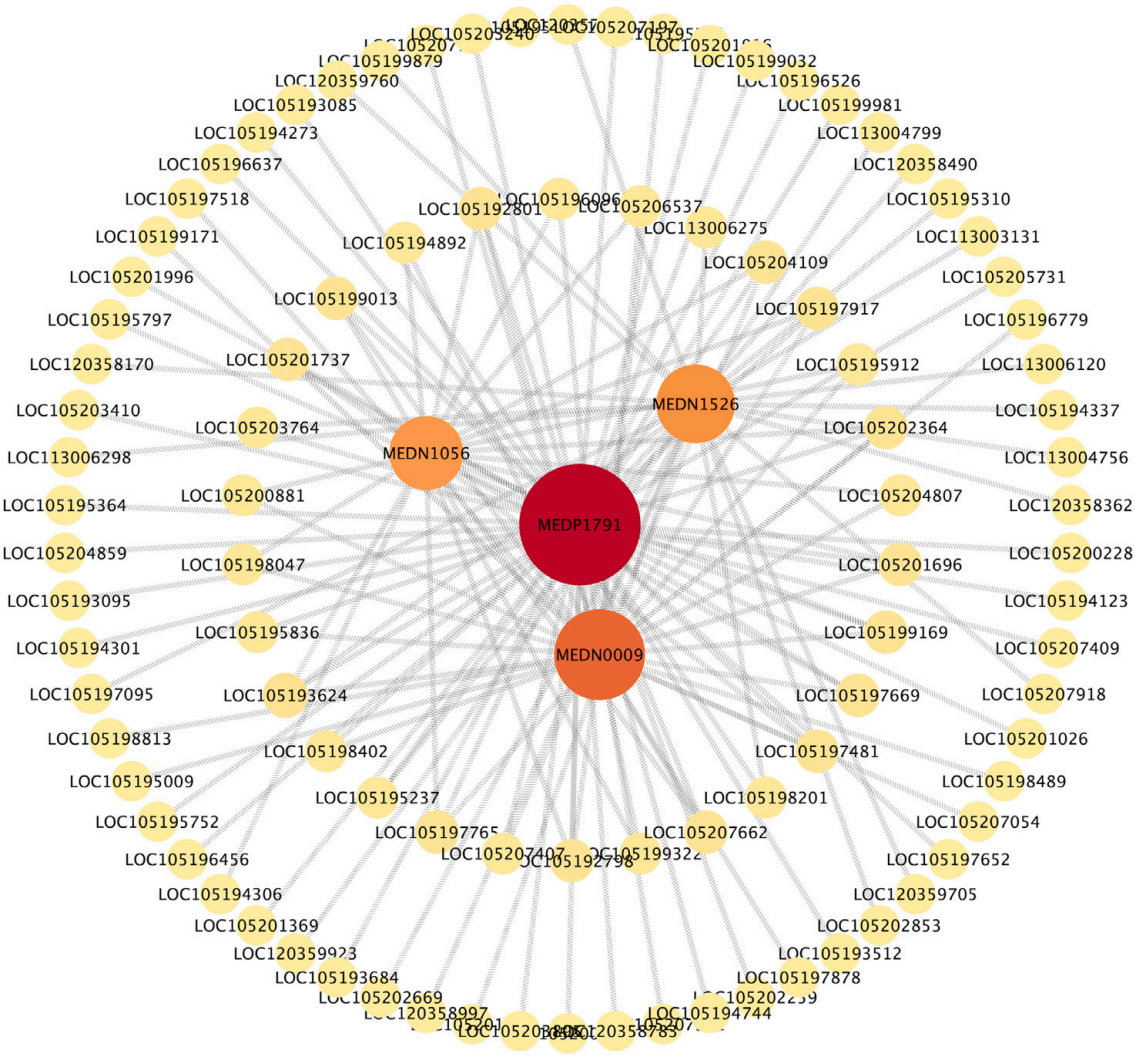
The correlation network showed connections between genes and metabolites in *Solenopsis invicta* post cycloxaprid exposure. The network indicated the complex relationships and potential interactions between the transcriptomic and metabolomic changes post cycloxaprid exposure.

Correlation analysis revealed a significant association between 226 DEGs and 54 DMs (|R| > 0.8, *p* < 0.05). The nine-quadrant diagram (Figure 7) showed that the regulatory relationships between DEGs and DMs changed over time post cycloxaprid exposure. There was a notably higher number of points in Quadrants 1 and 3 at 48 h (Figure 7C) compared with 12 and 24 h (Figures 7A,B), indicating a significant shift in the gene expression patterns that caused an increased number of metabolites influenced by both upregulated and downregulated DEGs. Meanwhile, the number of points in Quadrant 7 remained relatively stable across the time points, suggesting a persistent association between downregulated DEGs and decreased DMs throughout the experiment. In addition, we generated a series of correlation network diagrams (|R| > 0.85, *p* < 0.05) to provide a more extensive perspective on the interaction between DEGs and DMs in *S. invicta* post cycloxaprid exposure (Figure 8), which highlights the intricate relationships between the transcriptomic and metabolomic changes in response to cycloxaprid exposure.

## 4 Discussion

A few recent studies focused on the molecular effect of insecticides on *S. invica*. For example, Siddiqui et al. (2022a) utilized transcriptomic analysis to investigate the detoxification mechanisms of *S. invicta* in response to beta-cypermethrin and fipronil, revealing significant alterations in metabolic pathways, insulin signaling pathways, and fatty acid metabolism. This study also detected changes in the expression of detoxification genes within the families of acetylcholinesterase (AChE), carboxylesterase (CarE), glutathione S-transferase (GST), and cytochrome P450 (CYP450). Similarly, Siddiqui et al. (2022b) reported that indoxacarb treatment also affected these pathways and genes in *S. invicta*. In addition, Xiong et al. (2022) utilized qRT-PCR and RNA interference (RNAi) technologies to investigate the function of specific CYP450 family genes of *S. invicta* treated with fluralaner, and they found that several genes, such as *CYP9AS16*, *CYP6AS161*, *CYP6SQ20*, and *CYP336A45*, were closely related to the detoxification process of fluralaner. These findings align with the present study, providing a consistent view of the insecticide response at the molecular level in *S. invicta*.

Cycloxaprid has recently garnered substantial attention as a promising alternative to traditional insecticides, owing to its potential to counter resistance issues for agricultural pests (Wang et al., 2018a; Cui et al., 2020). For example, Zhang et al. (2018) found a slower rate of resistance development to cycloxaprid in *Laodelphax striatellus* compared to chlorpyrifos and buprofezin. Remarkably, only a 10-fold increase in resistance was observed in an *L. striatellus* variant with cycloxaprid resistance, contrasting with the increases of 106-fold and 332-fold for buprofezin and chlorpyrifos, respectively, under equivalent selection pressures. Dong et al. (2022) revealed that an *A. gossypii* variant with cycloxaprid resistance demonstrated minimal cross-resistance to six of other insecticides. The fold increases in resistance were 4.3 for imidacloprid, 2.9 for acetamiprid, 3.7 for thiamethoxam, 6.1 for nitenpyram, 2.2 for flupyradifurone, and 4.5 for sulfoxaflor. Similarly, Cui et al. (2016) and Zhang et al. (2019) suggested no cross-resistance between cycloxaprid and imidacloprid in *A. gossypii* and *Nilaparvata lugens*. Some recent research has investigated the molecular effects of cycloxaprid on various sucking insect pests (Yang et al., 2016; Jin et al., 2017). For example, Jin et al. (2021) identified 22 genes (e.g., genes in the families of CYP450 and GST) potentially associated with *Sogatella furcifera*’s resistance to cycloxaprid using transcriptomic analysis. Zhang et al. (2018) found that the resistance to cycloxaprid in *L. striatellus* was linked to the modified expression of nicotinic acetylcholine receptor subunits. Moreover, they reported that a knockdown of the *Lsa1* gene significantly amplified the susceptibility of the cycloxaprid-resistant strain to cycloxaprid. While these insights shed light on the resistance mechanisms of sucking insects, it is crucial to note that successful control of eusocial pests, such as *S. invicta,* primarily depends on the slow-action and non-repellency of cycloxaprid.

In our study, transcriptomic analyses revealed alterations in various physiological processes of *S. invicta* post cycloxaprid exposure over different durations. At 12 h, most DEGs were upregulated and enriched pathways involved in fatty acid metabolism, fatty acid biosynthesis, AMPK signaling pathway, insulin signaling pathway, and acyltransferase activity. Fatty acids are essential for energy storage and release, cell membrane assembly, and the production of signaling molecules in insects (Canavoso et al., 2001; Arrese and Soulages, 2010). The insulin signaling pathway assumes a crucial role in sustaining energy homeostasis through regulating carbohydrate, lipid, protein, and energy metabolism in insects (Wu and Brown, 2006; Géminard et al., 2009; Nässel and Broeck, 2016), which promotes glucose uptake and glycolysis, stimulates lipogenesis, and suppresses lipolysis and gluconeogenesis (Broughton et al., 2005). Acyltransferases are critical in synthesizing and degrading various lipids, including fatty acids, glycerolipids, and phospholipids (Van der Horst, 2003; Arrese and Soulages, 2010). In addition, the AMPK is activated under energy-deprived conditions (e.g., low ATP levels) and can initiate various downstream processes that help maintain energy balance by regulating fatty acid synthesis, oxidation, and other related processes (Pan and Hardie, 2002; Hardie, 2007; Sinnett and Brenman, 2016). Changes in these pathways collectively point to an enhancement in lipid and energy metabolism in *S. invicta*. Neonicotinoid insecticides can bind to nAChRs located in the central nervous systems, leading to excitation, convulsions, and death of insects, accompanied by increased energy consumption (Matsuda et al., 2001; Tomizawa and Casida, 2003; Jeschke et al., 2011). It is probable that *S. invicta* actively enhanced its lipid metabolism to generate more energy to compensate for the increased energy expenditure after cycloxaprid exposure, thereby maintaining survival and recovery.

At 24 h, most DEGs were upregulated and enriched in pathways involved with protein digestion and absorption, pancreatic secretion, proteolysis, and amino sugar and nucleotide sugar metabolism. The upregulation of genes involved in protein digestion and absorption may suggest increased efficiency in breaking down dietary proteins and assimilating amino acids (Holtof et al., 2019). This process could be essential for maintaining energy balance because amino acids can be an energy source when required (MacFarlane, 2018). Similarly, the upregulation of genes involved in pancreatic secretion and proteolysis indicates the enhanced capacity to produce and secrete digestive enzymes, which are crucial for breaking down proteins into smaller peptides and amino acids (van Hoef et al., 2011). These results strongly correlate with findings at 12 h, indicating that *S. invicta* may enhance its protein and carbohydrate metabolism to obtain more energy to support the heightened energy consumption post cycloxaprid exposure.

At 48 h, most DEGs were downregulated and enriched in pathways of fatty acid metabolism, fatty acid biosynthesis, AMPK signaling, and insulin signaling. This may indicate a significant reduction in lipid and energy metabolism of *S. invicta* at the later stage after cycloxaprid exposure. Furthermore, the downregulation of genes involved in iron ion and heme binding suggests a decreased ability to bind and utilize iron and heme. This could impact various biological processes such as oxygen transport, energy production, and DNA synthesis (Nichol et al., 2002; Walter-Nuno et al., 2013). Upregulated pathways mainly include oxidoreductase activity, transmembrane transport, extracellular region, and cAMP signaling, implying the increased oxidative stress response that may alter membrane transport, enhance extracellular communication, and modulate intracellular signaling cascades (Kunieda et al., 2006; Holtof et al., 2019; Wu et al., 2019; Liu et al., 2021).

Interestingly, 22 DEGs were enriched in pathways related to odorant perception at 12 h. *Solenopsis invicta* typically detects odor molecules through olfactory receptors located on their antennae. The alterations in these genes suggest a disturbance of olfactory perception in *S. invicta*, which may ultimately cause reduced sensitivity to cycloxaprid. Additional researches are required in the future to examine the potential involvement of these genes in the non-repellent effect of cycloxaprid on *S. invicta*. We also investigated the expression of detoxification-related genes, and identified 22 CYP450 family genes and 2 GST family genes. At 12 h, *LOC105207882* and *LOC120359407* were upregulated, suggesting that ants may actively adjust their detoxification mechanisms to counteract the effects of cycloxaprid during early exposure. At 24 h, the detoxification response seems to weaken because only one gene (*LOC105206318*) was upregulated, whereas four genes (*LOC105198272*, *LOC105203688*, *LOC120357488*, and *LOC120359317*) were downregulated. This shift indicates that *S. invicta* may experience increased stress due to continued exposure to cycloxaprid, thereby hindering their ability to maintain effective detoxification processes. At 48 h, 18 detoxification DEGs were downregulated, and only two genes (*LOC105205661* and *LOC105206584*) were upregulated, indicating that *S. invicta* may be unable to sustain its detoxification capacity after prolonged exposure, which may lead to impaired physiological function and eventually death. It is worth noting that the expression changes in the CYP450 family and GST family genes could have interconnected impacts on other physiological processes, such as lipid metabolism, energy metabolism, and immune function. For instance, at 12 h, the activation of these genes may necessitate energy, thus promoting lipid and energy metabolism. However, at 48 h, the decline in lipid and energy metabolism may contribute to the inability of these genes to maintain the function of detoxification.

Metabolomic analyses showed the diversity and functions of DMs in *S. invicta* following exposure to cycloxaprid over different durations. These DMs are various fatty acids, nucleotides, hormones, and amines, which participate in numerous physiological processes, including lipid and energy metabolism, DNA synthesis, hormone signaling, neurotransmission, and overall metabolic activity (Weyel and Wegener, 1996; Gäde et al., 2003; Kaczmarek and Boguś, 2021; Barbero et al., 2023). Correlation analysis showed that 226 DEGs exhibited significant correlations with 54 DMs (|R| > 0.8, *p* < 0.05), indicating the tight interplay between metabolites and genes in *S. invicta* treated with cycloxaprid. The correlation network (Figure 8) showed that the nicotinamide riboside (NR, MEDP1791) was strongly interconnected with numerous metabolites and genes. NR is a pyridine-nucleoside and a form of vitamin B_3_ and serves as an essential precursor to nicotinamide adenine dinucleotide (NAD^+^) (Bogan and Brenner, 2008), which is a cofactor for enzymes involved in energy metabolism and various metabolic pathways such as glycolysis and fatty acid oxidation (Xie et al., 2020). Therefore, NR might contribute to the adaptive responses of *S. invicta* to cycloxaprid, and future studies are needed to verify this.

Notably, we found that changes in fatty acids were inversely correlated with the expression levels of genes implicated in fatty acid synthesis and metabolism pathways. Transcriptomic data revealed upregulation of DEGs associated with fatty acid pathways at 12 h and downregulation at 48 h. In contrast, metabolomic analyses detected only two upregulated fatty acids at 12 h, and eight carnitines were still upregulated at 48 h. Carnitine is an endogenous compound carrier molecule in lipid metabolism and energy homeostasis (Bremer, 1983; Bieber, 1988; Pande and Murthy, 1989). The diminished lipid and energy metabolism in *S. invicta* may disrupt fatty acid cycling, resulting in carnitine accumulation at 48 h. Numerous studies involving humans and animals have demonstrated the beneficial effects of carnitine accumulation in treating diverse health conditions, such as arrhythmia, gastrointestinal discomfort, and epilepsy (Longo et al., 2006; Fukuda et al., 2015; Wang et al., 2018b). Concurrently, recent research suggests that the regulation of specific fatty acids might play a significant role in determining the potential of *Leptinotarsa decemlineata* to develop resistance to neonicotinoid insecticide (Clements et al., 2020). Therefore, the alterations in fatty acid (including carnitine) might also enhance the capacity of *S. invicta* to adapt to cycloxaprid. It is important to note that the present study was conducted using *S. invicta* workers, and future studies would be valuable to assess the impacts of cycloxaprid on different developmental stages of *S. invicta*.

## 5 Conclusion

In conclusion, our study provides a comprehensive understanding of the slow-acting and non-repellent properties of cycloxaprid against *S. invicta*. The slow-acting nature of cycloxaprid can be ascribed to the dynamic physiological responses observed in *S. invicta* post exposure, encompassing the enhancement of lipid and energy metabolism, alterations in immune function, and adjustments in detoxification mechanisms. These enhancements enable *S. invicta* to maintain their survival and recovery during the initial stages of exposure to cycloxaprid. However, they are insufficient to completely escape the lethal threat posed by the insecticide. As exposure duration increases, cycloxaprid progressively impairs physiological function, ultimately leading to the ants’ death. Furthermore, the non-repellent characteristic of cycloxaprid is likely associated with changes in olfactory perception-related genes, which may induce a disorder in *S. invicta*’s olfactory perception, resulting in reduced sensitivity to cycloxaprid. Consequently, the ants do not avoid their counterparts contaminated with cycloxaprid, thereby enhancing the effectiveness of this insecticide in controlling *S. invicta* at colony levels.

## Data Availability

The original contributions presented in the study are included in the article/Supplementary Material. The datasets presented in this study can be found in online repositories. This data can be found in National Center for Biotechnology Information (NCBI) BioProject database under the accession number PRJNA1005227.

## References

[B1] Angulo E. Hoffmann B. D. Ballesteros-Mejia L. Taheri A. Balzani P. Bang A. (2022). Economic costs of invasive alien ants worldwide. Biol. Invasions 24 (7), 2041–2060. 10.1007/s10530-022-02791-w

[B2] Arrese E. L. Soulages J. L. (2010). Insect fat body: energy, metabolism, and regulation. Annu. Rev. Entomol. 55, 207–225. 10.1146/annurev-ento-112408-085356 19725772 PMC3075550

[B3] Ascunce M. S. Yang C.-C. Oakey J. Calcaterra L. Wu W.-J. Shih C.-J. (2011). Global invasion history of the fire ant *Solenopsis invicta* . Science 331 (6020), 1066–1068. 10.1126/science.1198734 21350177

[B4] Barbero F. Mannino G. Casacci L. P. (2023). The role of biogenic amines in social insects: with a special focus on ants. Insects 14, 386. 10.3390/insects14040386 37103201 PMC10142254

[B5] Bernaba M. Power E. Campion J. Gotzek D. Schmidt J. O. Klotz S. A. (2019). Unconscious woman in shock and covered with ants pulled from an abandoned automobile. Am. J. Med. 132 (10), 1239–1241. 10.1016/j.amjmed.2019.02.053 30953629

[B6] Bieber L. L. (1988). Carnitine. Annu. Rev. Biochem. 57 (1), 261–283. 10.1146/annurev.bi.57.070188.001401 3052273

[B7] Bogan K. L. Brenner C. (2008). Nicotinic acid, nicotinamide, and nicotinamide riboside: a molecular evaluation of NAD^+^ precursor vitamins in human nutrition. Annu. Rev. Nutr. 28, 115–130. 10.1146/annurev.nutr.28.061807.155443 18429699

[B8] Bremer J. (1983). Carnitine--metabolism and functions. Physiol. Rev. 63 (4), 1420–1480. 10.1152/physrev.1983.63.4.1420 6361812

[B9] Broughton S. J. Piper M. D. W. Ikeya T. Bass T. M. Jacobson J. Driege Y. (2005). Longer lifespan, altered metabolism, and stress resistance in *Drosophila* from ablation of cells making insulin-like ligands. P. Natl. Acad. Sci. U. S. A. 102 (8), 3105–3110. 10.1073/pnas.0405775102 PMC549445 15708981

[B10] Buchfink B. Xie C. Huson D. (2015). Fast and sensitive protein alignment using DIAMOND. Nat. Methods 12, 59–60. 10.1038/nmeth.3176 25402007

[B11] Canavoso L. E. Jouni Z. E. Karnas K. J. Pennington J. E. Wells M. A. (2001). Fat metabolism in insects. Annu. Rev. Nutr. 21 (1), 23–46. 10.1146/annurev.nutr.21.1.23 11375428

[B12] Chan K. H. Guénard B. (2020). Ecological and socio-economic impacts of the red import fire ant, *Solenopsis invicta* (Hymenoptera: Formicidae), on urban agricultural ecosystems. Urban Ecosyst. 23, 1–12. 10.1007/s11252-019-00893-3

[B13] Chen J. (2007). Advancement on techniques for the separation and maintenance of the red imported fire ant colonies. Insect Sci. 14 (1), 1–4. 10.1111/j.1744-7917.2007.00120.x

[B14] Chen J. Oi D. H. (2020). Naturally occurring compounds/materials as alternatives to synthetic chemical insecticides for use in fire ant management. Insects 11, 758. 10.3390/insects11110758 33158097 PMC7694179

[B15] Chen J. Zhou Y. Lei Y. Shi Q. Qi G. He Y. (2022). Role of the foraging gene in worker behavioral transition in the red imported fire ant, *Solenopsis invicta* (Hymenoptera: Formicidae). Pest Manag. Sci. 78 (7), 2964–2975. 10.1002/ps.6921 35419943

[B16] Chen S. Zhou Y. Chen Y. Gu J. (2018). fastp: an ultra-fast all-in-one FASTQ preprocessor. Bioinformatics 34, i884–i890. 10.1093/bioinformatics/bty560 30423086 PMC6129281

[B17] Chen W. Gong L. Guo Z. Wang W. Zhang H. Liu X. (2013). A novel integrated method for large-scale detection, identification, and quantification of widely targeted metabolites: application in the study of rice metabolomics. Mol. Plant 6 (6), 1769–1780. 10.1093/mp/sst080 23702596

[B18] Clements J. Olson J. M. Sanchez-Sedillo B. Bradford B. Groves R. L. (2020). Changes in emergence phenology, fatty acid composition, and xenobiotic-metabolizing enzyme expression is associated with increased insecticide resistance in the Colorado potato beetle. Arch. Insect Biochem. 103 (3), e21630. 10.1002/arch.21630 PMC7027459 31621115

[B19] Cui L. Qi H. Yang D. Yuan H. Rui C. (2016). Cycloxaprid: a novel *cis*-nitromethylene neonicotinoid insecticide to control imidacloprid-resistant cotton aphid (*Aphis gossypii*). Pestic. Biochem. Phys. 132, 96–101. 10.1016/j.pestbp.2016.02.005 27521919

[B20] Cui L. Sun L. Yang D. Yan X. Yuan H. (2012). Effects of cycloxaprid, a novel *cis*-nitromethylene neonicotinoid insecticide, on the feeding behaviour of *Sitobion avenae* . Pest Manag. Sci. 68, 1484–1491. 10.1002/ps.3333 22707457

[B21] Cui L. Wang Q. Wang Q. Wang L. Yuan H. Rui C. (2020). Cycloxaprid: a novel *cis*-nitromethylene neonicotinoid insecticide to control *Bemisia tabaci* . Pest Manag. Sci. 76 (5), 1705–1712. 10.1002/ps.5693 31758644

[B22] Cui L. Yuan H. Wang Q. Wang Q. Rui C. (2018). Sublethal effects of the novel *cis*-nitromethylene neonicotinoid cycloxaprid on the cotton aphid *Aphis gossypii* Glover (Hemiptera: aphididae). Sci. Rep. 8, 8915. 10.1038/s41598-018-27035-7 29891984 PMC5995959

[B23] Dong W. Yang H. Wang C. Li H. Shang J. Chen Z. (2022). Cross-resistance and fitness costs of the *cis*-nitromethylene neonicotinoid cycloxaprid resistance in melon aphid, *Aphis gossypii* (Hemiptera: aphididae). J. Econ. Entomol. 115 (5), 1668–1675. 10.1093/jee/toac112 35899798

[B24] Drees B. M. Gold R. E. (2003). Development of integrated pest management programs for the red imported fire ant (Hymenoptera: Formicidae). J. Entomol. Sci. 38 (2), 170–180. 10.18474/0749-8004-38.2.170

[B25] Fukuda M. Kawabe M. Takehara M. Iwano S. Kuwabara K. Kikuchi C. (2015). Carnitine deficiency: risk factors and incidence in children with epilepsy. Brain Dev. 37 (8), 790–796. 10.1016/j.braindev.2014.12.004 25547040

[B26] Gäde G. Goldsworthy G. J. Gäde G. Goldsworthy G. J. Gäde G. Goldsworthy G. J. (2003). Insect peptide hormones: a selective review of their physiology and potential application for pest control. Pest Manag. Sci. 59, 1063–1075. 10.1002/ps.755 14561063

[B27] Géminard C. Rulifson E. J. Léopold P. (2009). Remote control of insulin secretion by fat cells in *Drosophila* . Cell Metab. 10 (3), 199–207. 10.1016/j.cmet.2009.08.002 19723496

[B28] Gordon D. M. (2019). The ecology of collective behavior in ants. Annu. Rev. Entomol. 64, 35–50. 10.1146/annurev-ento-011118-111923 30256667

[B29] Gutrich J. J. VanGelder E. Loope L. (2007). Potential economic impact of introduction and spread of the red imported fire ant, *Solenopsis invicta*, in Hawaii. Environ. Sci. Policy 10 (7-8), 685–696. 10.1016/j.envsci.2007.03.007

[B30] Haddad Junior V. Larsson C. E. (2015). Anaphylaxis caused by stings from the *Solenopsis invicta*, lava-pés ant or red imported fire ant. An. Bras. Dermatol. 90, 22–25. 10.1590/abd1806-4841.20153420 26312665 PMC4540499

[B31] Hardie D. G. (2007). AMP-activated protein kinase as a drug target. Annu. Rev. Pharmacol. 47, 185–210. 10.1146/annurev.pharmtox.47.120505.105304 16879084

[B32] Haynes W. (2013). “Benjamini–hochberg method,” in Encyclopedia of systems biology. Editors Dubitzky W. Wolkenhauer O. Cho K. H. Yokota H. (New York, NY: Springer). 10.1007/978-1-4419-9863-7_1215

[B33] Holtof M. Lenaerts C. Cullen D. Vanden Broeck J. (2019). Extracellular nutrient digestion and absorption in the insect gut. Cell Tissue Res. 377, 397–414. 10.1007/s00441-019-03031-9 31037358

[B34] Jeschke P. Nauen R. Schindler and M. Elbert A. (2011). Overview of the status and global strategy for neonicotinoids. J. Agr. Food Chem. 59 (7), 2897–2908. 10.1021/jf101303g 20565065

[B35] Jin J.-X. Jin D.-C. Li F.-L. Cheng Y. Li W.-H. Ye Z.-C. (2017). Expression differences of resistance-related genes induced by cycloxaprid using qRT-PCR in the female adult of *Sogatella furcifera* (Hemiptera: Delphacidae). J. Econ. Entomol. 110 (4), 1785–1793. 10.1093/jee/tox155 28854654

[B36] Jin J.-X. Ye Z.-C. Jin D.-C. Li F.-L. Li W.-H. Cheng Y. (2021). Changes in transcriptome and gene expression in *Sogatella furcifera* (Hemiptera: Delphacidae) in response to cycloxaprid. J. Econ. Entomol. 114 (1), 284–297. 10.1093/jee/toaa238 33151323

[B37] Kaczmarek A. Boguś M. (2021). The metabolism and role of free fatty acids in key physiological processes in insects of medical, veterinary and forensic importance. PeerJ 9, e12563. 10.7717/peerj.12563 35036124 PMC8710053

[B38] Kim D. Langmead B. Salzberg S. L. (2015). HISAT: a fast spliced aligner with low memory requirements. Nat. Methods 12 (4), 357–360. 10.1038/nmeth.3317 25751142 PMC4655817

[B39] Kohlmeier P. Negroni M. A. Kever M. Emmling S. Stypa H. Feldmeyer B. (2017). Intrinsic worker mortality depends on behavioral caste and the queens’ presence in a social insect. Sci. Nat. 104, 34. 10.1007/s00114-017-1452-x 28353195

[B40] Kunieda T. Fujiyuki T. Kucharski R. Foret S. Ament S. A. Toth A. L. (2006). Carbohydrate metabolism genes and pathways in insects: insights from the honey bee genome. Insect Mol. Biol. 15 (5), 563–576. 10.1111/j.1365-2583.2006.00677.x 17069632 PMC1847477

[B41] Liao Y. Smyth G. K. Shi W. (2014). featureCounts: an efficient general purpose program for assigning sequence reads to genomic features. Bioinformatics 30 (7), 923–930. 10.1093/bioinformatics/btt656 24227677

[B42] Liu N. Wang Y. Li T. Feng X. (2021). G-protein coupled receptors (GPCRs): signaling pathways, characterization, and functions in insect physiology and toxicology. Int. J. Mol. Sci. 22 (10), 5260. 10.3390/ijms22105260 34067660 PMC8156084

[B43] Livak K. J. Schmittgen T. D. (2001). Analysis of relative gene expression data using real-time quantitative PCR and the 2^−ΔΔCT^ Method. Methods 25 (4), 402–408. 10.1006/meth.2001.1262 11846609

[B44] Longo N. Amat di San Filippo C. Pasquali M. (2006). Disorders of carnitine transport and the carnitine cycle. Am. J. Med. Genet. 142C (2), 77–85. 10.1002/ajmg.c.30087 16602102 PMC2557099

[B45] Love M. I. Huber W. Anders S. (2014). Moderated estimation of fold change and dispersion for RNA-seq data with DESeq2. Genome Biol. 15 (12), 550. 10.1186/s13059-014-0550-8 25516281 PMC4302049

[B46] MacFarlane N. G. (2018). Digestion and absorption. Anaesth. Intensive Care Med. 19 (3), 125–127. 10.1016/j.mpaic.2018.01.001

[B47] Matsuda K. Buckingham S. D. Kleier D. Rauh J. J. Grauso M. Sattelle D. B. (2001). Neonicotinoids: insecticides acting on insect nicotinic acetylcholine receptors. Trends Pharmacol. Sci. 22 (11), 573–580. 10.1016/S0165-6147(00)01820-4 11698101

[B48] Morrison L. W. (2002). Long-term impacts of an arthropod-community invasion by the imported fire ant, *Solenopsis invicta* . Ecology 83, 2337–2345. 10.1890/0012-9658(2002)083[2337:ltioaa]2.0.co;2

[B49] Nässel D. R. Broeck J. V. (2016). Insulin/IGF signaling in *Drosophila* and other insects: factors that regulate production, release and post-release action of the insulin-like peptides. Cell Mol. Life Sci. 73, 271–290. 10.1007/s00018-015-2063-3 26472340 PMC11108470

[B50] Negroni M. A. Macit M. N. Stoldt M. Feldmeyer B. Foitzik S. (2021). Molecular regulation of lifespan extension in fertile ant workers. Phil. Trans. R. Soc. B 376, 20190736. 10.1098/rstb.2019.0736 33678017 PMC7938160

[B51] Nichol H. Law J. H. Winzerling J. J. (2002). Iron metabolism in insects. Annu. Rev. Entomol. 47 (1), 535–559. 10.1146/annurev.ento.47.091201.145237 11729084

[B52] Ning D. Yang F. Xiao Q. Ran H. Xu Y. (2019). A simple and efficient method for preventing ant escape (Hymenoptera: Formicidae). Myrmecol. News 29, 57–65. 10.25849/myrmecol.news_029:057

[B53] Pan D. A. Hardie D. G. (2002). A homologue of AMP-activated protein kinase in *Drosophila melanogaster* is sensitive to AMP and is activated by ATP depletion. Biochem. J. 367 (1), 179–186. 10.1042/bj20020703 12093363 PMC1222868

[B54] Pande S. V. Murthy M. S. R. (1989). Carnitine: vitamin for an insect, vital for man. Biochem. Cell Biol. 67 (10), 671–673. 10.1139/o89-100 2686718

[B55] Pereira R. M. Williams D. F. Becnel J. J. Oi D. H. (2002). Yellow-head disease caused by a newly discovered *Mattesia* sp. in populations of the red imported fire ant, *Solenopsis invicta* . J. Invertebr. Pathol. 81, 45–48. 10.1016/S0022-2011(02)00116-7 12417212

[B56] Pertea M. Kim D. Pertea G. M. Leek J. T. Salzberg S. L. (2016). Transcript-level expression analysis of RNA-seq experiments with HISAT, StringTie and Ballgown. Nat. Protoc. 11 (9), 1650–1667. 10.1038/nprot.2016.095 27560171 PMC5032908

[B57] Robinson G. E. (1992). Regulation of division of labor in insect societies. Annu. Rev. Entomol. 37 (1), 637–665. 10.1146/annurev.en.37.010192.003225 1539941

[B58] Robinson M. D. McCarthy D. J. Smyth G. K. (2010). edgeR: a Bioconductor package for differential expression analysis of digital gene expression data. Bioinformatics 26 (1), 139–140. 10.1093/bioinformatics/btp616 19910308 PMC2796818

[B59] Rust M. K. Su N.-Y. (2012). Managing social insects of urban importance. Annu. Rev. Entomol. 57, 355–375. 10.1146/annurev-ento-120710-100634 21942844

[B60] Shannon P. Markiel A. Ozier O. Baliga N. S. Wang J. T. Ramage D. (2003). Cytoscape: a software environment for integrated models of biomolecular interaction networks. Genome Res. 13 (11), 2498–2504. 10.1101/gr.1239303 14597658 PMC403769

[B61] Shao X. Liu Z. Xu X. Li Z. Qian X. (2013a). Overall status of neonicotinoid insecticides in China: production, application and innovation. J. Pestic. Sci. 38, 1–9. 10.1584/jpestics.D12-037

[B62] Shao X. Swenson T. L. Casida J. E. (2013b). Cycloxaprid insecticide: nicotinic acetylcholine receptor binding site and metabolism. J. Agric. Food Chem. 61, 7883–7888. 10.1021/jf4030695 23889077

[B63] Siddiqui J. A. Luo Y. Sheikh U. A. A. Bamisile B. S. Khan M. M. Imran M. (2022a). Transcriptome analysis reveals differential effects of beta-cypermethrin and fipronil insecticides on detoxification mechanisms in *Solenopsis invicta* . Front. Physiol. 13, 1018731. 10.3389/fphys.2022.1018731 36277215 PMC9583148

[B64] Siddiqui J. A. Zhang Y. Luo Y. Bamisile B. S. Rehman N. U. Islam W. (2022b). Comprehensive detoxification mechanism assessment of red Imported fire ant (*Solenopsis invicta*) against Indoxacarb. Molecules 27, 870. 10.3390/molecules27030870 35164134 PMC8839056

[B65] Silverman J. Brightwell R. J. (2008). The argentine ant: challenges in managing an invasive unicolonial pest. Annu. Rev. Entomol. 53, 231–252. 10.1146/annurev.ento.53.103106.093450 17877449

[B66] Sinnett S. E. Brenman J. E. (2016). The role of AMPK in *Drosophila melanogaster* . AMP-Activated protein kinase 107, 389–401. 10.1007/978-3-319-43589-3_16 PMC5835264 27812989

[B67] Tomizawa M. Casida J. E. (2003). Selective toxicity of neonicotinoids attributable to specificity of insect and mammalian nicotinic receptors. Annu. Rev. Entomol. 48 (1), 339–364. 10.1146/annurev.ento.48.091801.112731 12208819

[B68] Van der Horst D. J. (2003). Insect adipokinetic hormones: release and integration of flight energy metabolism. Comp. Biochem. Physiology Part B 136 (2), 217–226. 10.1016/S1096-4959(03)00151-9 14529748

[B69] Van Hoef V. Breugelmans B. Spit J. Simonet G. Zels S. Billen J. (2011). Functional analysis of a pancreatic secretory trypsin inhibitor-like protein in insects: silencing effects resemble the human pancreatic autodigestion phenotype. Insect biochem. molec. 41 (9), 688–695. 10.1016/j.ibmb.2011.04.012 21571068

[B70] Vinson S. B. (2013). Impact of the invasion of the imported fire ant. Insect Sci. 20 (4), 439–455. 10.1111/j.1744-7917.2012.01572.x 23955940

[B71] Walter-Nuno A. B. Oliveira M. P. Oliveira M. F. Gonçalves R. L. Ramos I. B. Koerich L. B. (2013). Silencing of maternal heme-binding protein causes embryonic mitochondrial dysfunction and impairs embryogenesis in the blood sucking insect *Rhodnius prolixus* . J. Biol. Chem. 288 (41), 29323–29332. 10.1074/jbc.M113.504985 23986441 PMC3795234

[B72] Wang L. Zeng L. Xu Y. Lu Y. (2020). Prevalence and management of *Solenopsis invicta* in China. Neobiota 54, 89–124. 10.3897/neobiota.54.38584

[B73] Wang R. Fang Y. Mu C. Qu C. Li F. Wang Z. (2018a). Baseline susceptibility and cross-resistance of cycloxaprid, a novel *cis*-nitromethylene neonicotinoid insecticide, in Bemisia tabaci MED from China. Crop Prot. 110, 283–287. 10.1016/j.cropro.2017.02.012

[B74] Wang R. Liu Y.-Y. Liu G.-H. Lu H.-B. Mao C.-Y. (2018b). l-Carnitine and heart disease. Life Sci. 194, 88–97. 10.1016/j.lfs.2017.12.015 29241711

[B75] Wen C. Shen L. Chen J. Zhang J. Feng Y. Wang Z. (2022). Red imported fire ants cover the insecticide-treated surfaces with particles to reduce contact toxicity. J. Pest Sci. 95 (3), 1135–1150. 10.1007/s10340-021-01474-0

[B76] Wetterer J. K. (2013). Exotic spread of *Solenopsis invicta* buren (hymenoptera: Formicidae) beyond north America. Sociobiology 60 (1), 50–55. 10.13102/sociobiology.v60i1.50-55

[B77] Weyel W. Wegener G. (1996). Adenine nucleotide metabolism during anoxia and postanoxic recovery in insects. Experientia 52, 474–480. 10.1007/BF01919319

[B78] Wu C. Chakrabarty S. Jin M. Liu K. Xiao Y. (2019). Insect ATP-binding cassette (ABC) transporters: roles in xenobiotic detoxification and bt insecticidal activity. Int. J. Mol. Sci. 20 (11), 2829. 10.3390/ijms20112829 31185645 PMC6600440

[B79] Wu Q. Brown M. R. (2006). Signaling and function of insulin-like peptides in insects. Annu. Rev. Entomol. 51, 1–24. 10.1146/annurev.ento.51.110104.151011 16332201

[B80] Wylie F. R. Janssen-May S. (2017). Red imported fire ant in Australia: what if we lose the war? Ecol. Manag. Restor. 18, 32–44. 10.1111/emr.12238

[B81] Xie N. Zhang L. Gao W. Huang C. Huber P. E. Zhou X. (2020). NAD^+^ metabolism: pathophysiologic mechanisms and therapeutic potential. Sig. Transduct. Target. Ther. 5, 227–237. 10.1038/s41392-020-00311-7 PMC7539288 33028824

[B82] Xiong T. Ling S. Liu J. Zeng X. (2022). Insecticidal and *P450* mediate metabolism of fluralaner against red imported fire ant, *Solenopsis invicta* (Hymenoptera: Formicidae). Pestic. Biochem. Physiol. 187, 105184. 10.1016/j.pestbp.2022.105184 36127046

[B83] Yang Y. Zhang Y. Yang B. Fang J. Liu Z. (2016). Transcriptomic responses to different doses of cycloxaprid involved in detoxification and stress response in the whitebacked planthopper, *Sogatella furcifera* . Entomol. Exp. Appl. 158 (3), 248–257. 10.1111/eea.12406

[B84] Zhang L. Wang L. Chen J. Zhang J. He Y. Lu Y. (2022). Toxicity, horizontal transfer, and physiological and behavioral effects of cycloxaprid against *Solenopsis invicta* (Hymenoptera: Formicidae). Pest Manag. Sci. 78 (6), 2228–2239. 10.1002/ps.6847 35192738

[B85] Zhang Y. Han Y. Yang Q. Wang L. He P. Liu Z. (2018). Resistance to cycloxaprid in *Laodelphax striatellus* is associated with altered expression of nicotinic acetylcholine receptor subunits. Pest Manag. Sci. 74 (4), 837–843. 10.1002/ps.4757 28991400

[B86] Zhang Y. Xu X. Bao H. Shao X. Li Z. Liu Z. (2019). The binding properties of cycloxaprid on insect native nAChRs partially explain the low cross-resistance with imidacloprid in *Nilaparvata lugens* . Pest Manag. Sci. 75 (1), 246–251. 10.1002/ps.5108 29877026

