# Supplementary material for: Transcriptome and metabolome comprehensive analysis reveal the molecular basis of slow-action and non-repellency of cycloxaprid against an eusocial pest, Solenopsis invicta
Source: Front Physiol. 2023 Nov 27;14:1274416. doi: 10.3389/fphys.2023.1274416 (PMC10711210; doi:10.3389/fphys.2023.1274416)
Supplement: Supplementary file 1 [file DataSheet1.docx]

**Table S1** Sequences of primers used for qRT-PCR analysis of selected genes

| **Genes** | **Forward primers (5'-3')** | **Reverse primers (5'-3')** |
| --- | --- | --- |
| *GST* | GCTATCCTGCGATAAGCGGT | GACTTCCGGGCGATTGGTAA |
| *AASS* | CCAGTACGTTGGTCAGAGGG | TTCACCATACCAGCGACACC |
| *CYP450-1* | TGGCGGAATTAATCGGAGCAT | TTTCGAAGCCAGCGAGGAAT |
| *ORAI* | TGCAATACGGAAGCACACGA | AAGCGATGAGCAGAGGATCG |
| *CYP450-2* | GGCTGGAAATTTGCCACGTT | TTGCTGGGATCTTCGTGCAT |
| *SCD* | TAAAGCCAGGCAACCACGAA | TGGTATGACAAACGTGCGGA |
| *ACO* | CGCGTCCGCACTTAATTCTC | TACGCGCACCAAGGTATGTT |
| *CYP450-3* | ACGGACTTGTATGCCGTGTT | CAGTCCATTATGTTTAAAGCTGTCG |
| *RPS* | GGAAGTCCGCGTTACTCCAA | ACGAGTGGCAACCTTCTCAG |

**Table S2** Sequencing quality control parameters

| **Sample** | **Raw Reads** | **Clean Reads** | **Clean Base (G)** | **Error Rate(%)** | **Q20 (%)** | **Q30 (%)** | **GC Content (%)** |
| --- | --- | --- | --- | --- | --- | --- | --- |
| CK_12h_1 | 52868194 | 43459576 | 6.52 | 0.03 | 96.04 | 90.38 | 46.66 |
| EG_12h_1 | 59977744 | 50539392 | 7.58 | 0.03 | 96.9 | 92.1 | 46.37 |
| CK_12h_2 | 60943716 | 51648386 | 7.75 | 0.03 | 97.11 | 92.4 | 46.87 |
| EG_12h_2 | 58296834 | 50346212 | 7.55 | 0.03 | 96.79 | 91.88 | 45.96 |
| CK_12h_3 | 57384810 | 49476170 | 7.42 | 0.03 | 97 | 92.18 | 46.59 |
| EG_12h_3 | 66502006 | 56609672 | 8.49 | 0.03 | 96.97 | 92.22 | 46.12 |
| CK_24h_1 | 57202364 | 48395510 | 7.26 | 0.03 | 97.2 | 92.58 | 46.8 |
| EG_24h_1 | 62855146 | 52907734 | 7.94 | 0.03 | 96.87 | 92.07 | 47.29 |
| CK_24h_2 | 59138902 | 51751636 | 7.76 | 0.03 | 96.88 | 92.06 | 46.55 |
| EG_24h_2 | 61542516 | 53530050 | 8.03 | 0.03 | 97.01 | 92.25 | 46.33 |
| CK_24h_3 | 65305812 | 55608626 | 8.34 | 0.03 | 96.91 | 92.16 | 47.36 |
| EG_24h_3 | 63474522 | 54506822 | 8.18 | 0.03 | 96.68 | 91.79 | 48.15 |
| CK_48h_1 | 59442530 | 50157490 | 7.52 | 0.03 | 96.71 | 91.77 | 44.85 |
| EG_48h_1 | 58934540 | 51333570 | 7.7 | 0.03 | 97.36 | 92.79 | 46.85 |
| CK_48h_2 | 61595414 | 54773074 | 8.22 | 0.03 | 97.38 | 92.81 | 46.08 |
| EG_48h_2 | 52364650 | 42596782 | 6.39 | 0.03 | 96.62 | 91.6 | 44.29 |
| CK_48h_3 | 60603116 | 51972796 | 7.8 | 0.03 | 97.1 | 92.4 | 46.7 |
| EG_48h_3 | 60328990 | 50206600 | 7.53 | 0.03 | 96.88 | 92.04 | 46.11 |


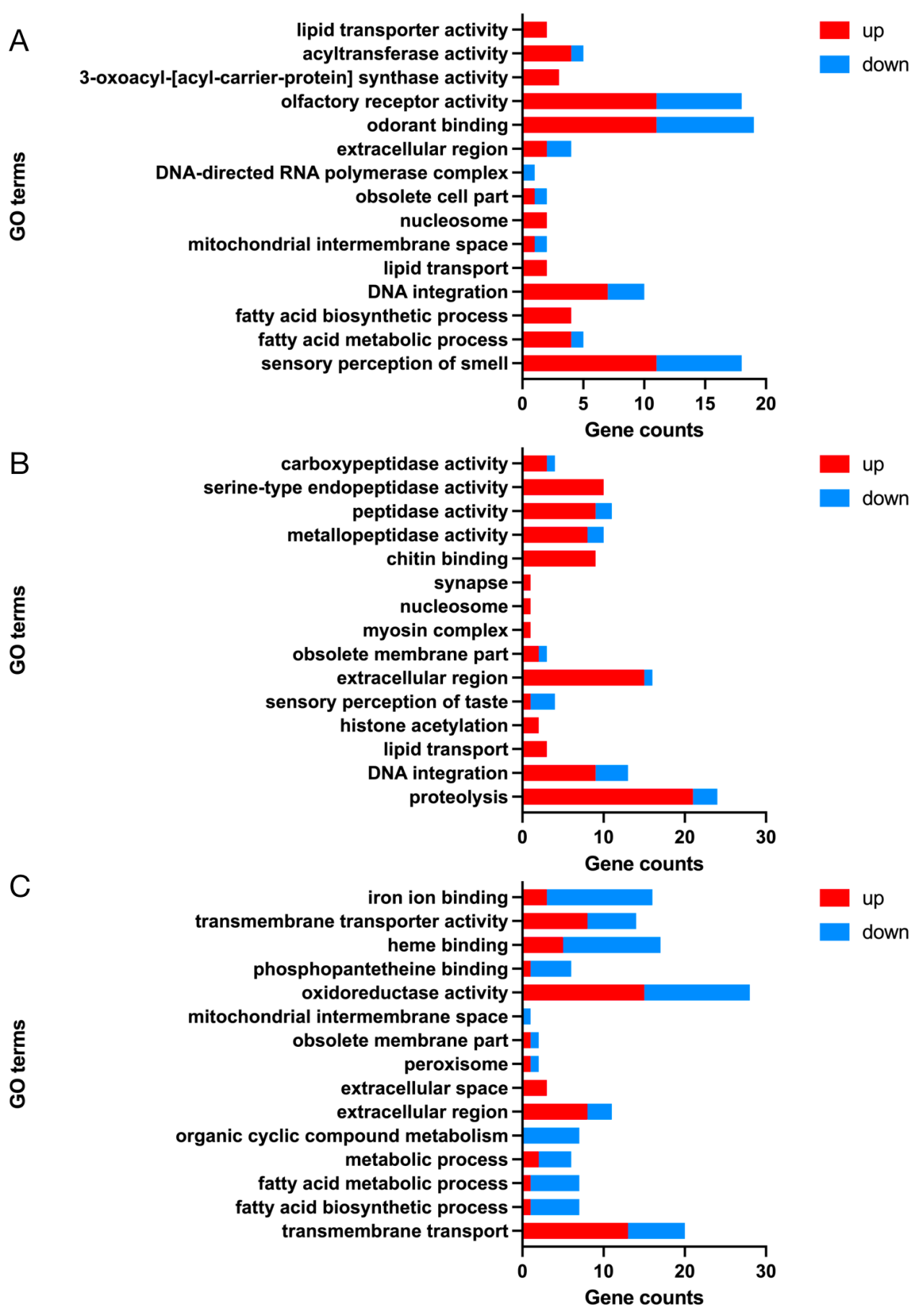


**Figure S1:** Regulation status of DEGs in significantly enriched GO terms post cycloxaprid exposure at 12 (A), 24 (B), and 48 h (C).


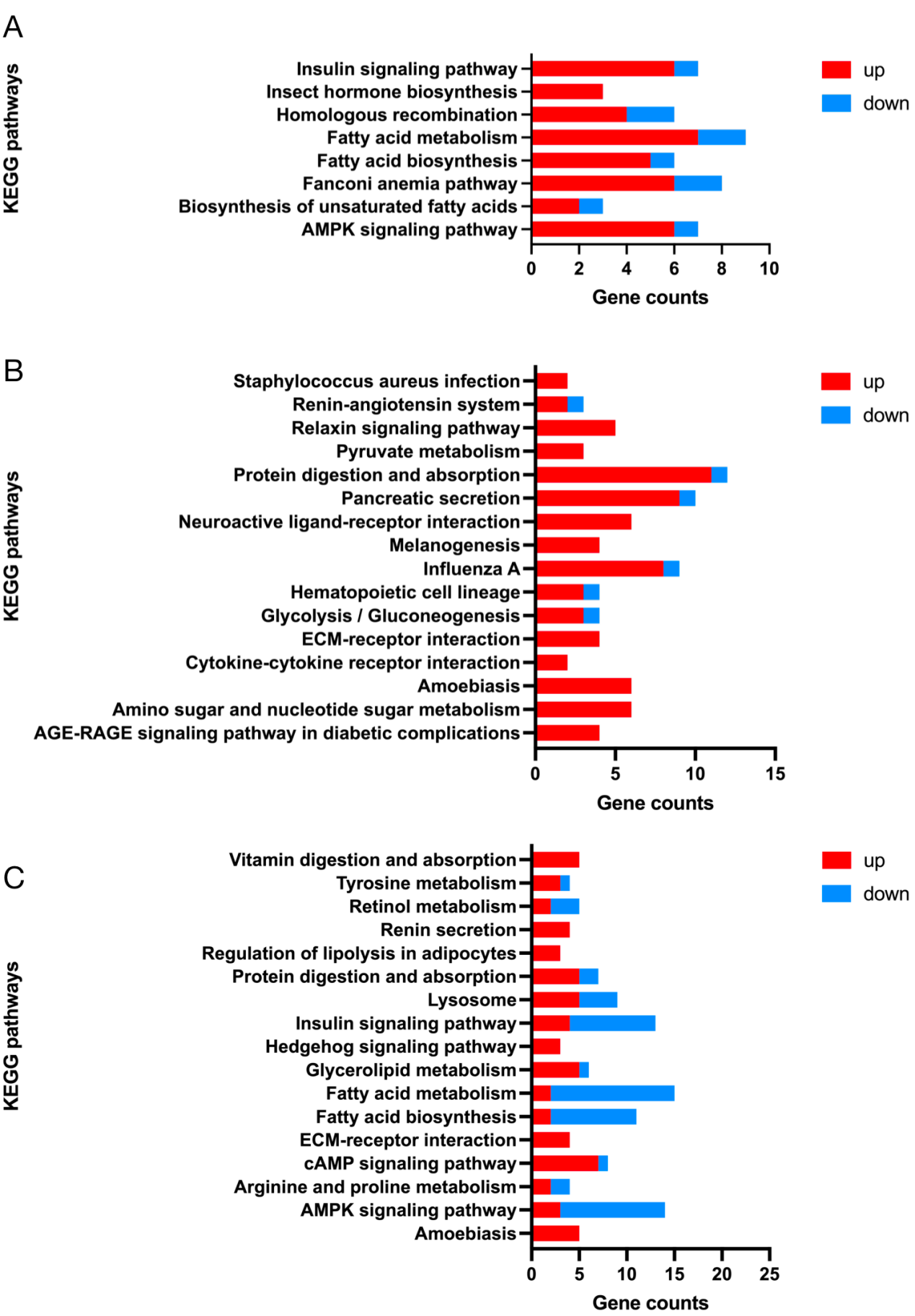


**Figure S2:** Regulation status of DEGs in significantly enriched KEGG pathways post cycloxaprid exposure at 12 (A), 24 (B), and 48 h (C).


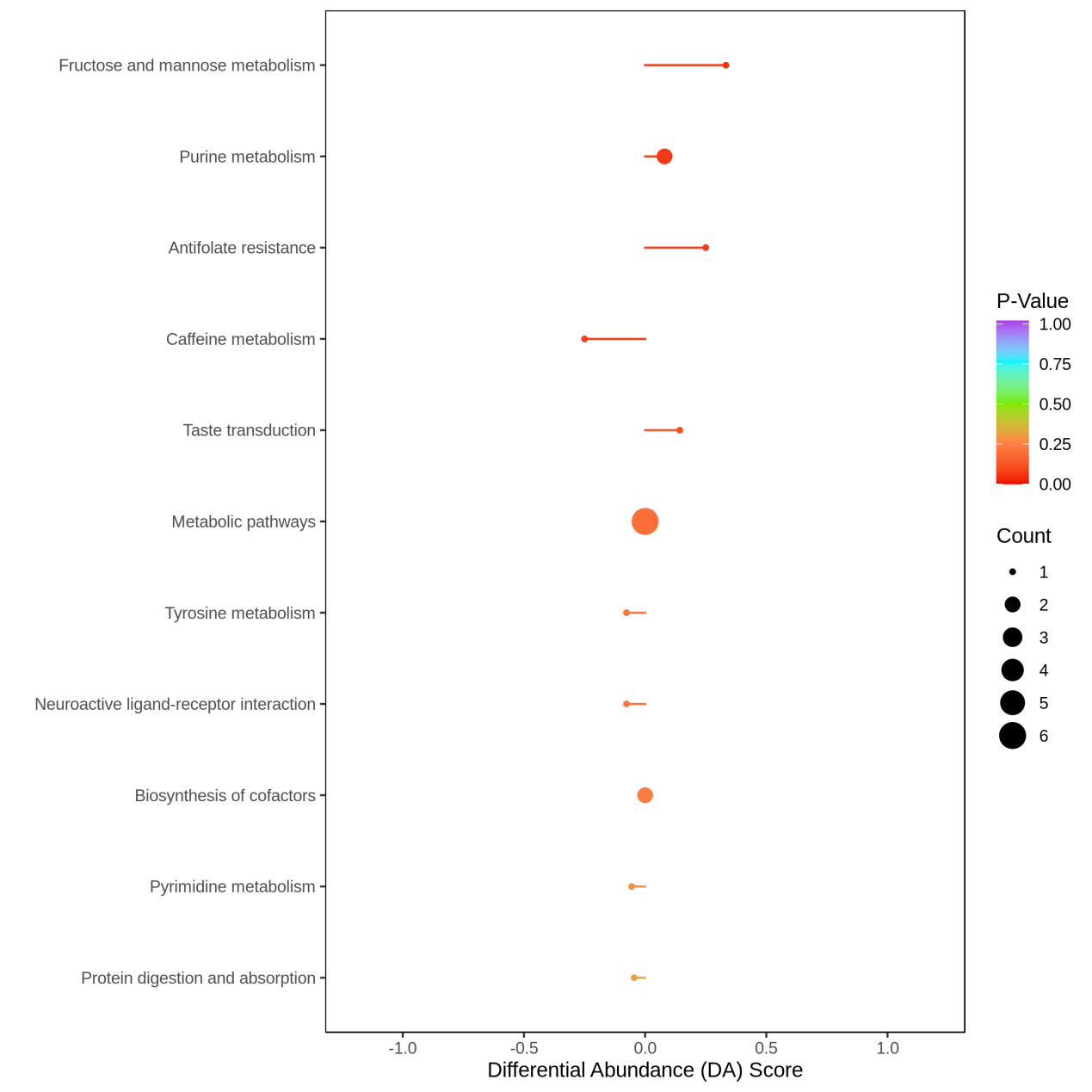


**Figure S3:** Discriminant Analysis (DA) score plot of KEGG enrichment analysis for DMs in *Solenopsis invicta* at 12 h post cycloxaprid exposure.


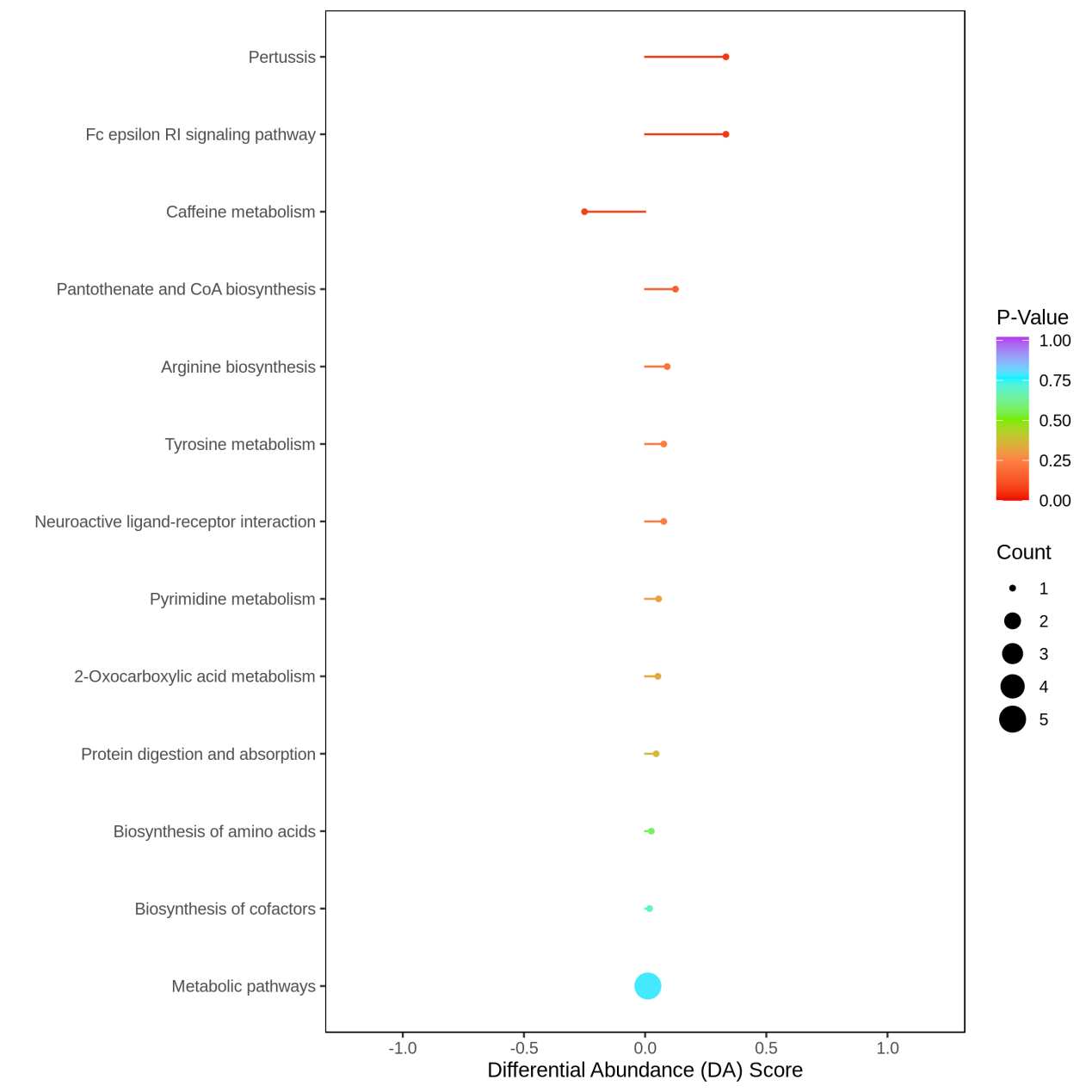


**Figure S4:** Discriminant Analysis (DA) score plot of KEGG enrichment analysis for DMs in *Solenopsis invicta* at 24 h post cycloxaprid exposure.


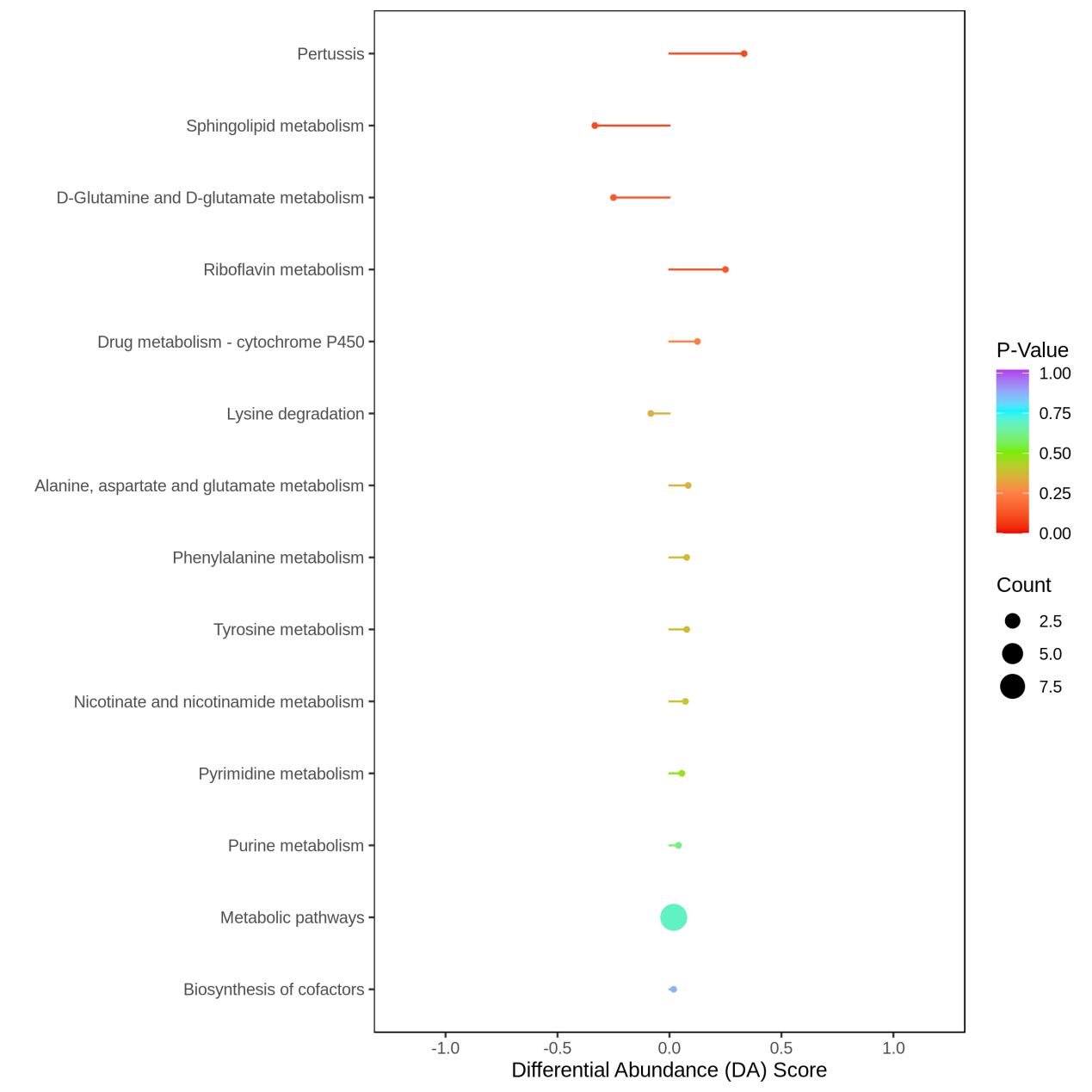


**Figure S5:** Discriminant Analysis (DA) score plot of KEGG enrichment analysis for DMs in *Solenopsis invicta* at 48 h post cycloxaprid exposure.
